# Supplementary material for: Pre-activated nanoparticles with persistent luminescence for deep tumor photodynamic therapy in gallbladder cancer
Source: Nat Commun. 2023 Sep 14;14:5699. doi: 10.1038/s41467-023-41389-1 (PMC10502062; doi:10.1038/s41467-023-41389-1)
Supplement: Supplementary file 1 — Supplementary Information [file 41467_2023_41389_MOESM1_ESM.docx]

**Supplementary Information**

**Pre-activated Nanoparticles with Persistent Luminescence for Deep Tumor Photodynamic Therapy in Gallbladder Cancer**

Sarun Juengpanich^1,2,3#^, Shijie Li^1,2#^, Taorui Yang^4#^, Tianao Xie^1,2^, Jiadong Chen^5^, Yukai Shan^1^, Jiyoung Lee^6^, Ziyi Lu^1^, Tianen Chen^1^, Bin Zhang^1^, Jiasheng Cao^1^, Jiahao Hu^1^, Jicheng Yu^1,6,7,8,9^, Yanfang Wang^6^, Win Topatana^1,2*^, Zhen Gu^1,6,7,8,9,10*^, Xiujun Cai^1,2,3*^, Mingyu Chen^1,2,3*^

1. Department of General Surgery, Sir Run-Run Shaw Hospital, Zhejiang University, Hangzhou 310016, China
2. School of Medicine, Zhejiang University, Hangzhou 310058, China
3. National Engineering Research Center of Innovation and Application of Minimally Invasive Instruments, Sir Run-Run Shaw Hospital, Zhejiang University, Hangzhou 310016, China
4. Department of Chemistry, Zhejiang Sci-Tech University, Hangzhou 310018, China
5. Department of Chemistry, Zhejiang University, Hangzhou 310016, China
6. Zhejiang Provincial Key Laboratory for Advanced Drug Delivery Systems, College of Pharmaceutical Sciences, Zhejiang University, Hangzhou, 310058, China
7. National Key Laboratory of Advanced Drug Delivery and Release Systems, Zhejiang University, Hangzhou, 310058, China
8. Liangzhu Laboratory, Zhejiang University Medical Center, Hangzhou, 311121, China
9. Jinhua Institute of Zhejiang University, Jinhua, 321299, China
10. MOE Key Laboratory of Macromolecular Synthesis and Functionalization, Department of Polymer Science and Engineering, Zhejiang University, Hangzhou, 310027, China

# These authors contributed equally to this work.

***Correspondence:** mychen@zju.edu.cn (M. Chen); srrsh_cxj@zju.edu.cn (X. Cai); guzhen@zju.edu.cn (Z. Gu); win.topatana@zju.edu.cn (W. Topatana)

**Supplementary Figures**


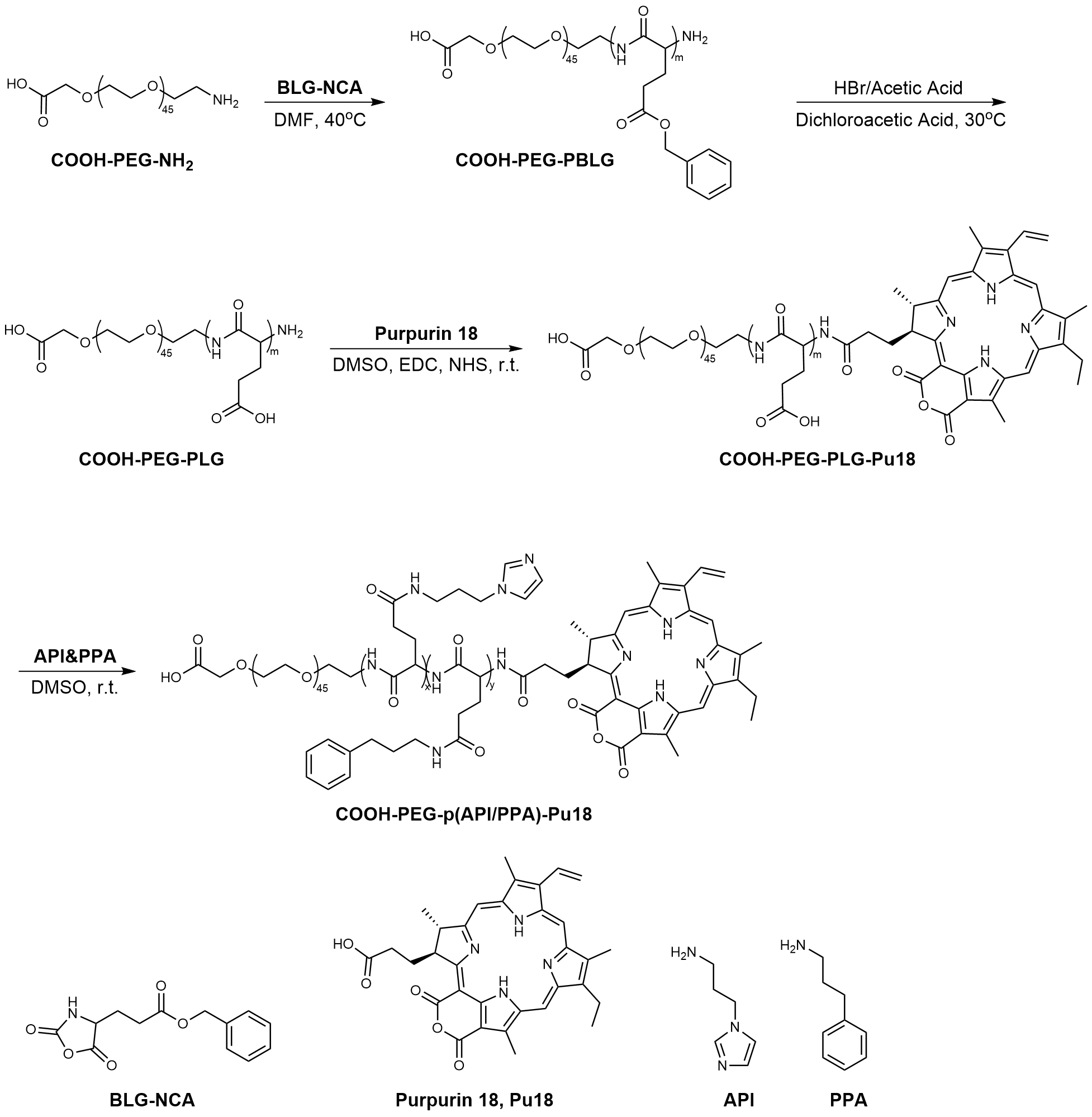


**Supplementary Figure 1:** Synthesis scheme of stimuli-responsive polymeric ligands (SPLs).


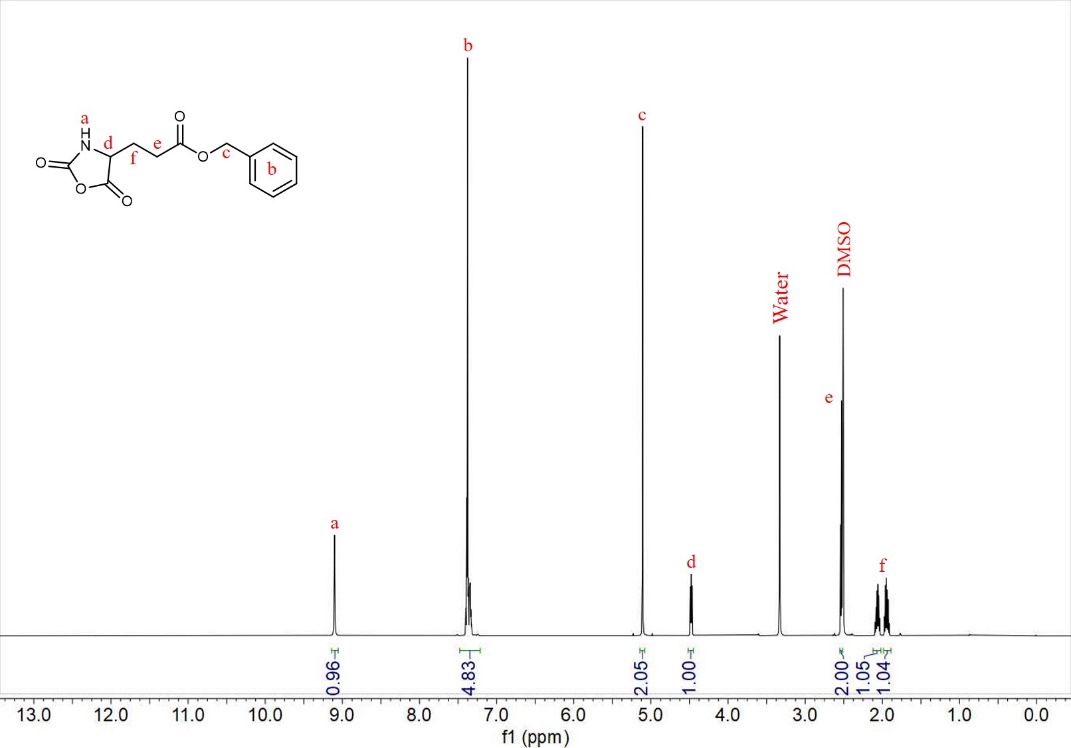


**Supplementary Figure 2:** ^1^H-NMR analysis of BLG-NCA.


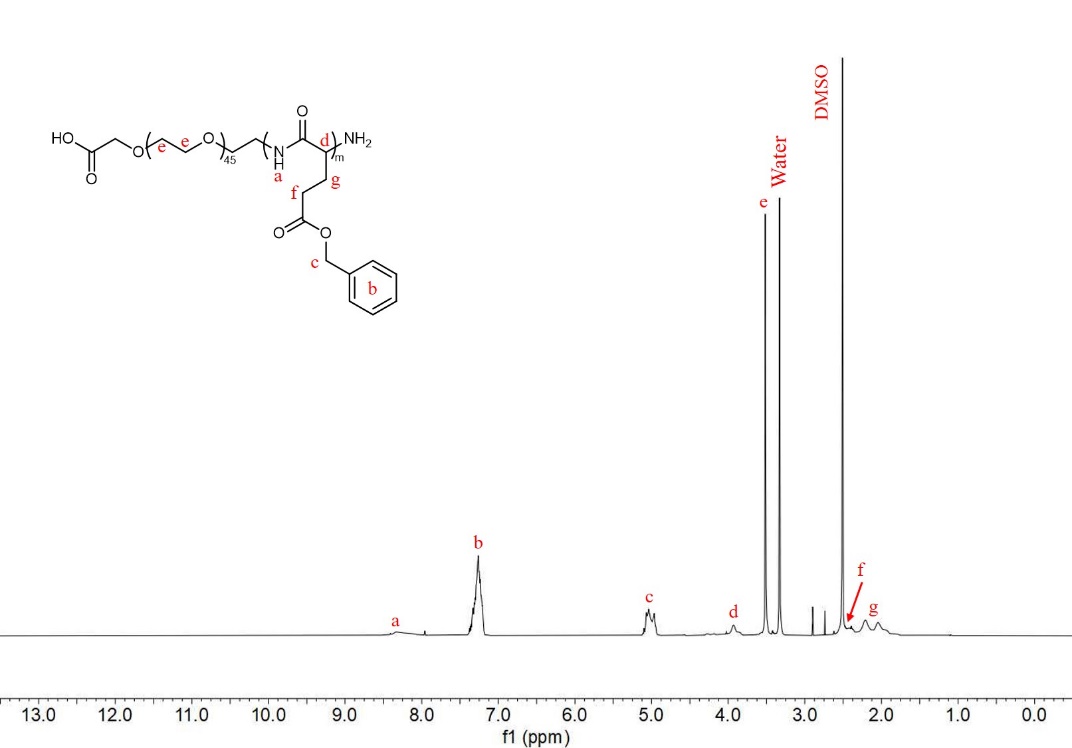


**Supplementary Figure 3:** ^1^H-NMR analysis of COOH-PEG-PBLG.


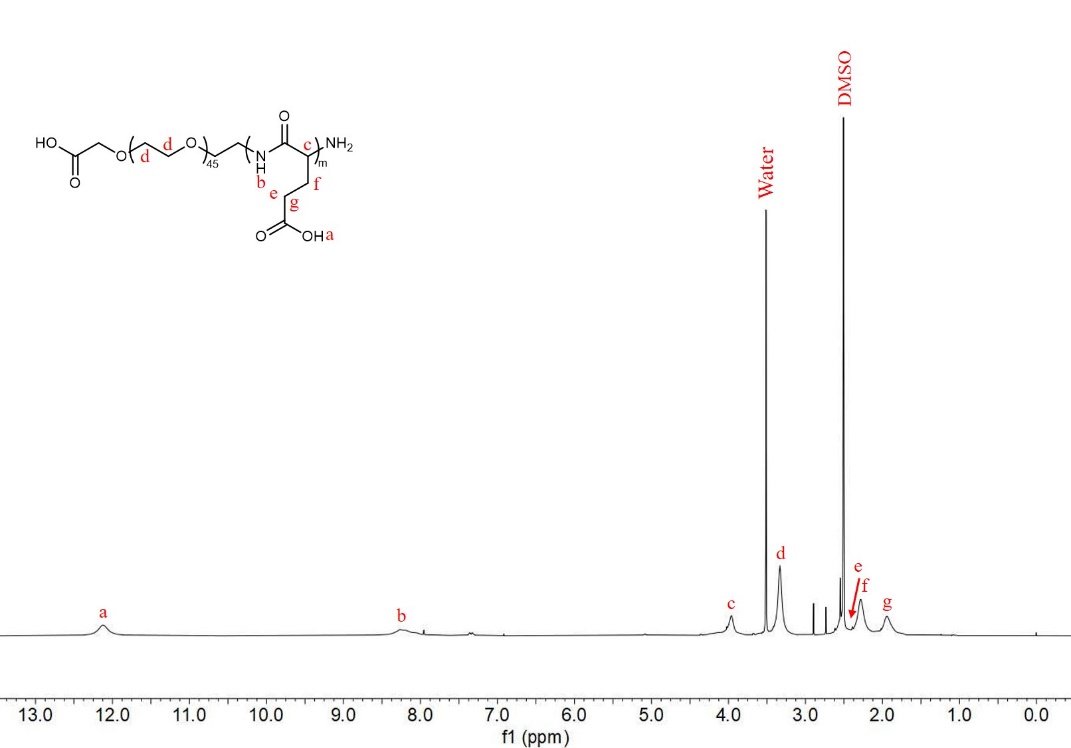


**Supplementary Figure 4:** ^1^H-NMR analysis of COOH-PEG-PLG.


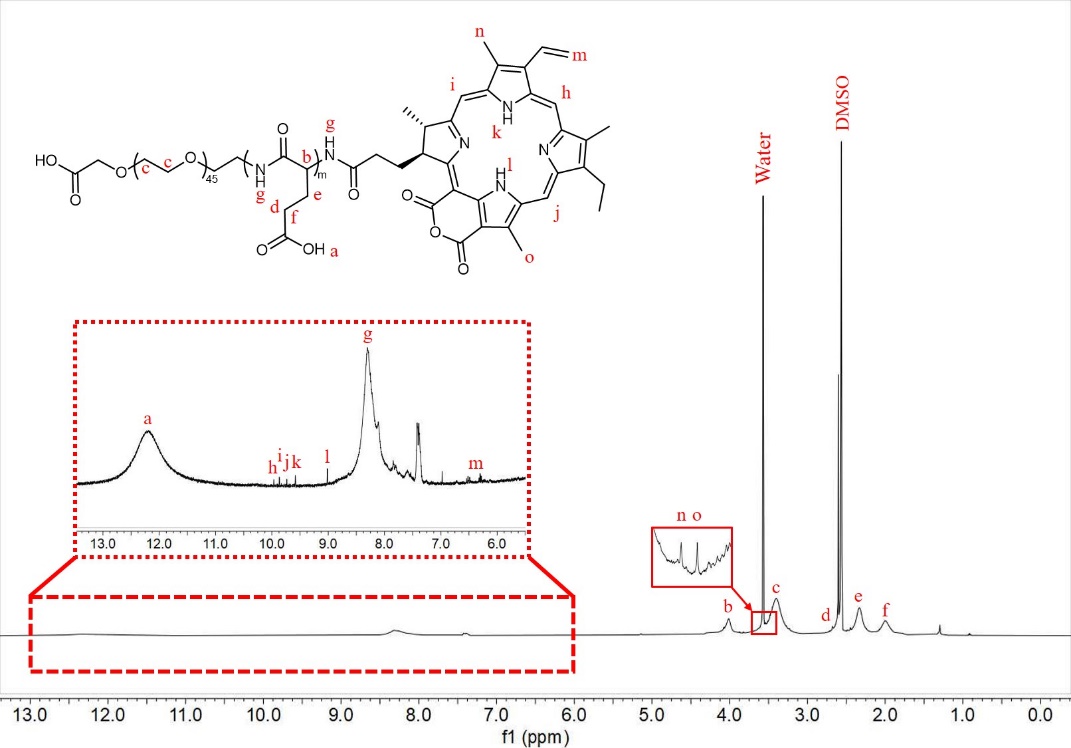


**Supplementary Figure 5:** ^1^H-NMR analysis of COOH-PEG-PLG-Pu18. The peaks at δ = 9.96, 9.86, and 9.73 ppm were attributed to the protons of porphyrin conjugate structure; δ = 9.58 and 9.01 ppm were attributed to the active protons of the porphyrin center; δ = 6.51 and 6.32 ppm were attributed to the protons of porphyrin terminal olefins; and δ = 3.87 and 3.82 ppm were attributed to the protons of porphyrin methyl. In addition, signals at δ = 4.01, 2.60, 2.33, and 1.99 ppm were attributed to the PLG chain protons and δ = 3.39 ppm were attributed to PEG.


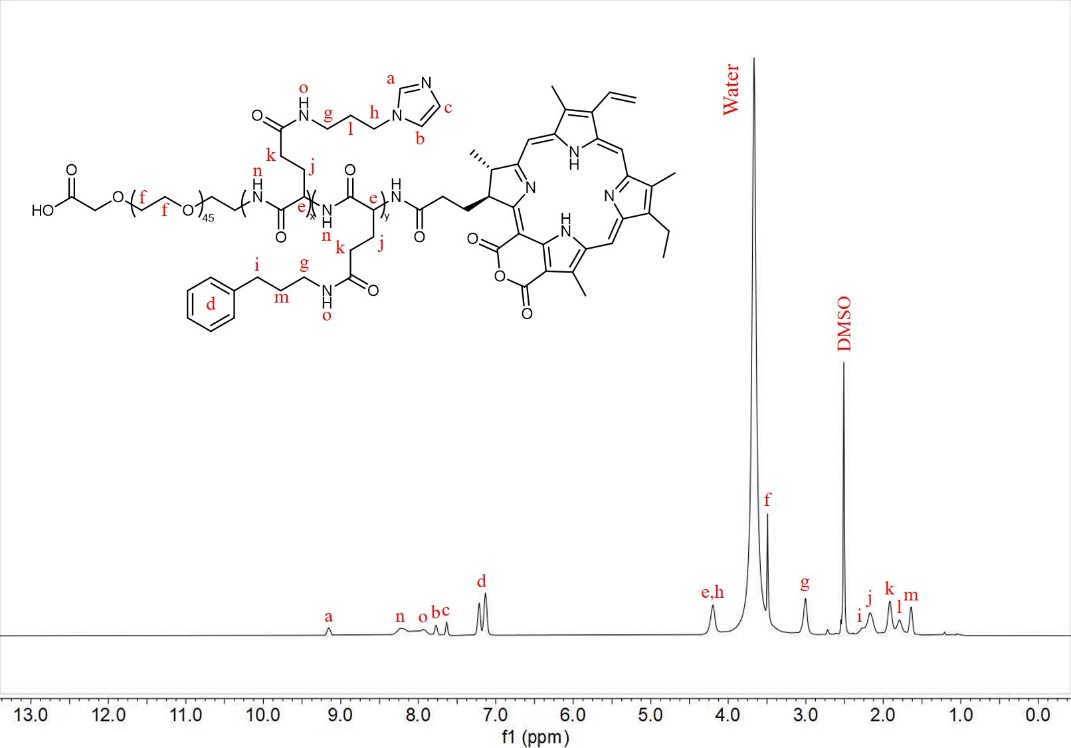


**Supplementary Figure 6:** ^1^H-NMR analysis of COOH-PEG-p(API/PPA)-Pu18. The peaks at δ = 9.15, 7.77, and 7.63 ppm were attributed to the protons of the API’s imidazole group, while the peaks at δ = 7.21 and 7.13 ppm were attributed to the phenyl protons of PPA.


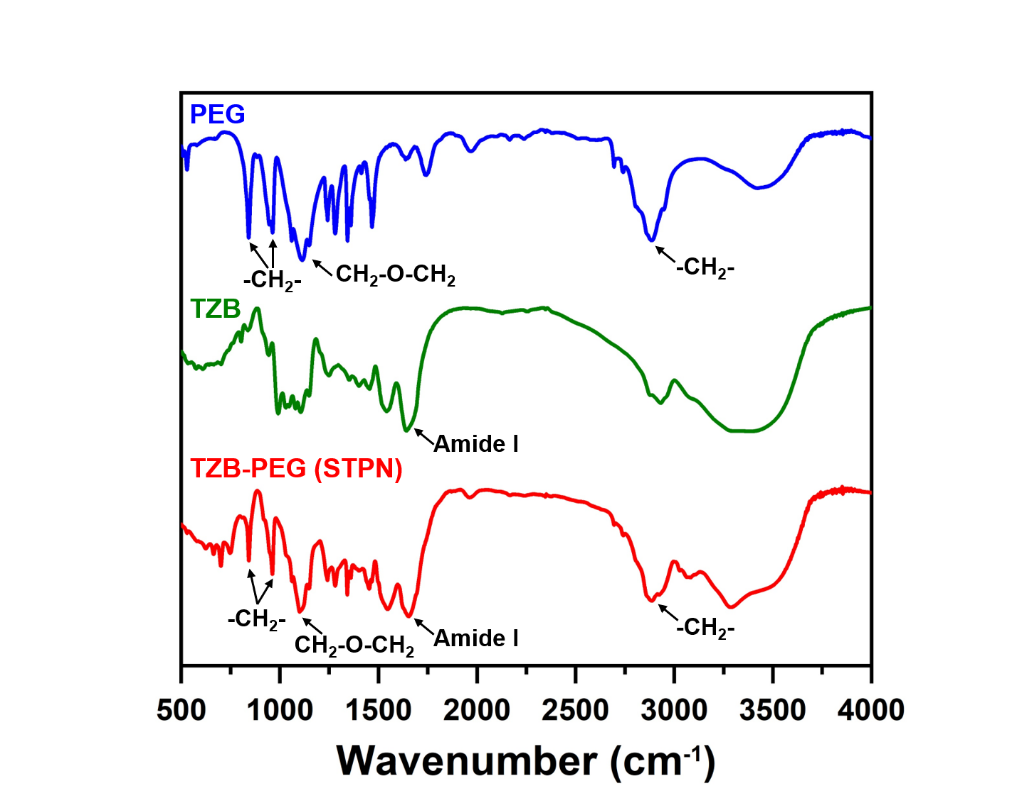


**Supplementary Figure 7:** FTIR analysis of TZB and PEG amine bond. The C-O stretching vibration and -CH_2_- rocking vibration at 2887 cm^-1^, 1110 cm^-1^, 962 cm^-1^, and 844 cm^-1^, as well as the C=O stretching vibration of amide I at 1645 cm^-1^ confirmed the successful conjugation between TZB and PEG.


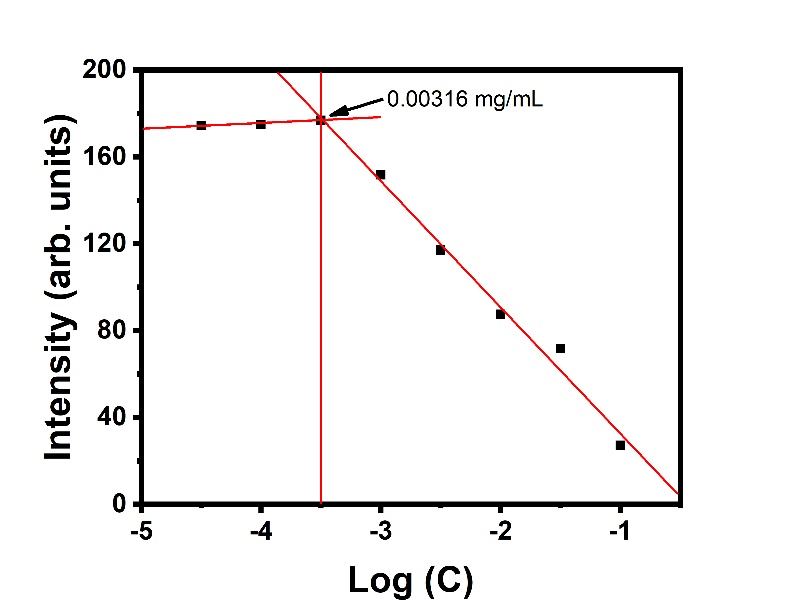


**Supplementary Figure 8:** Critical aggregation concentration (CAC) of COOH-PEG-p(API/PPA)-Pu18 (SPLs).


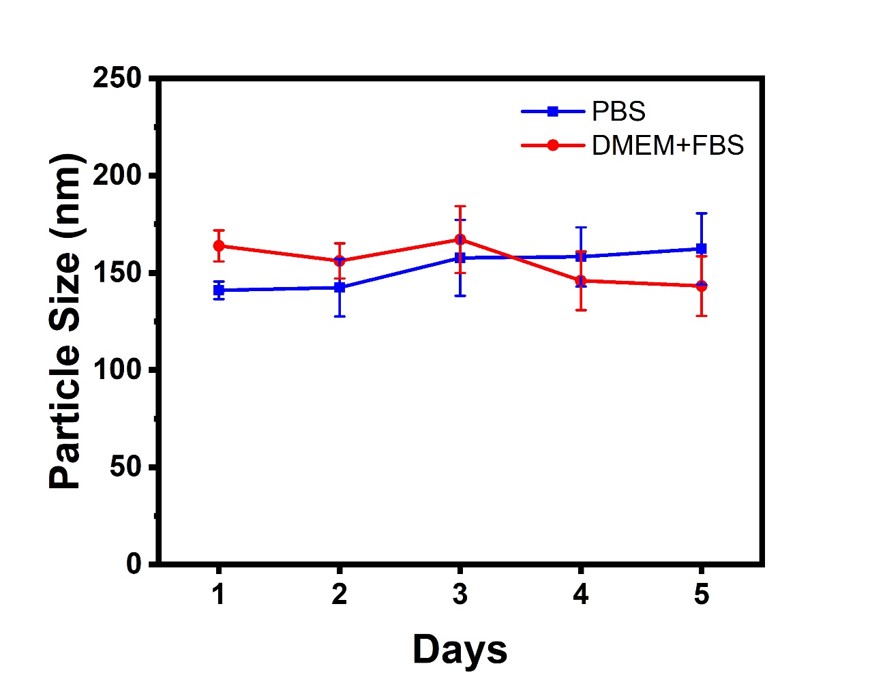


**Supplementary Figure 9:** Stability test of STPNs in PBS or cell culture medium via DLS measurements. The data are represented as mean ± SD (n = 3 independent experiments).


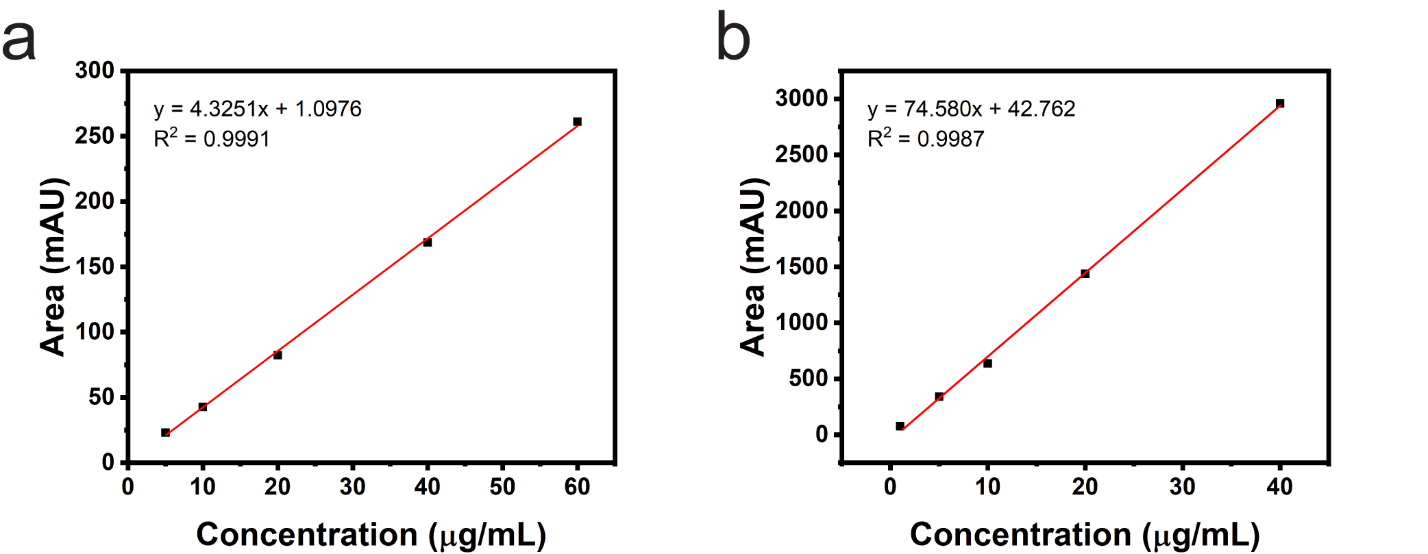


**Supplementary Figure 10:** HPLC calibration curve to calculate a) Trastuzumab and b) Purpurin 18 concentration in STPNs.


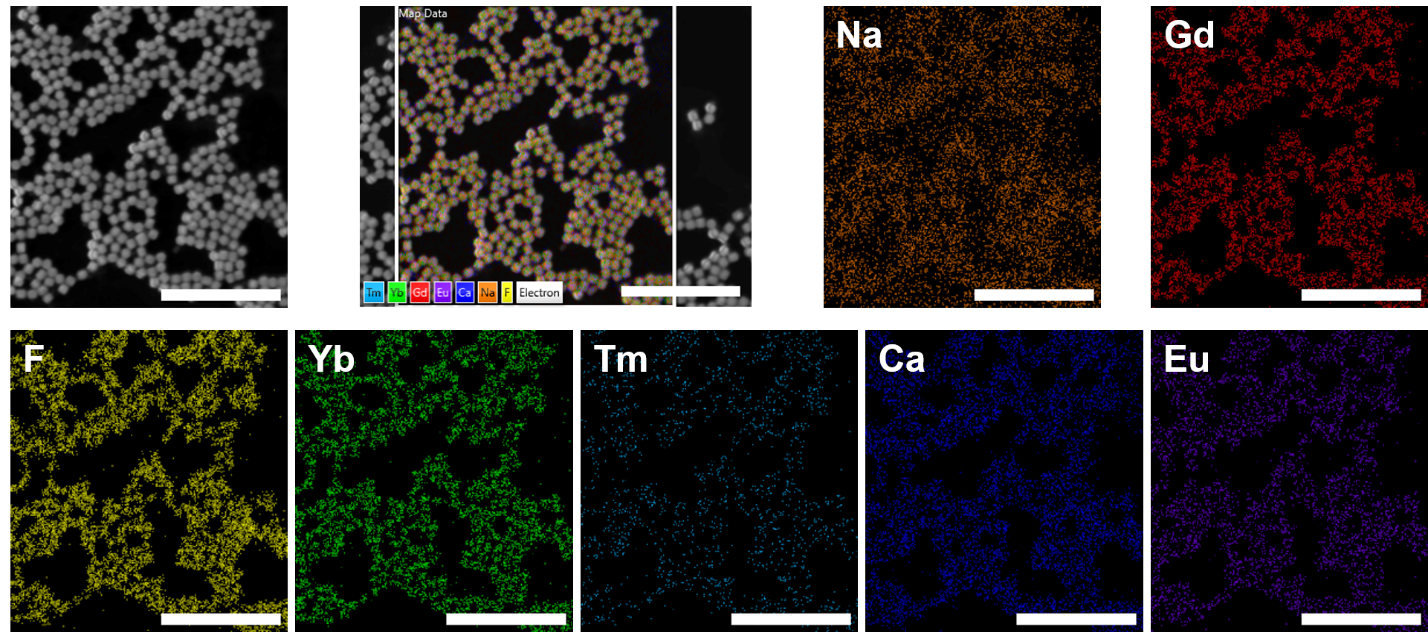


**Supplementary Figure 11:** NaGdF_4_:Yb,Tm@CaF_2_:Eu core@shell UCNPs HAADF imaging and EDS mapping (scale bar = 250 nm).


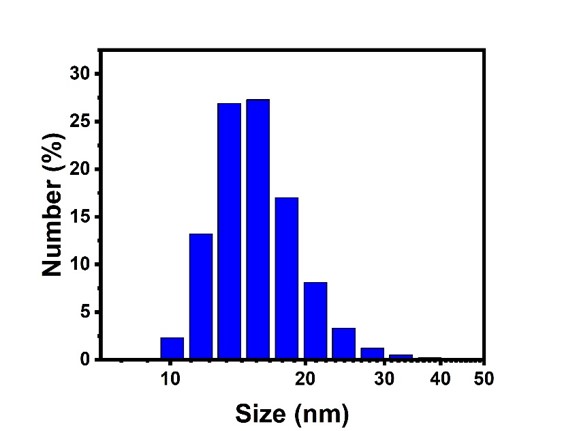


**Supplementary Figure 12:** DLS particle size distribution of NaGdF_4_:Yb,Tm@CaF_2_:Eu UCNPs.


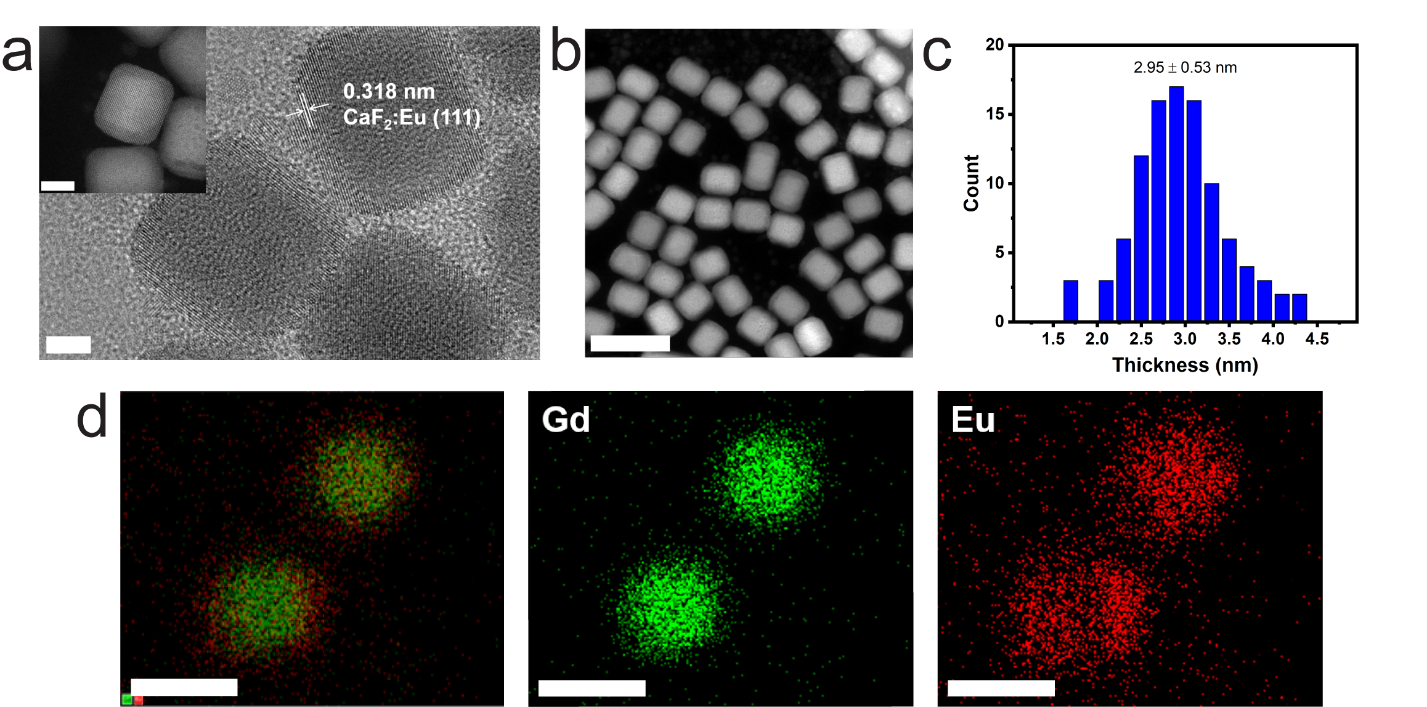


**Supplementary Figure 13:** Characterization of CaF_2_ shell morphology and thickness. a) HR-TEM imaging (scale bar = 5 nm) and b) HAADF-STEM imaging of NaGdF_4_:Yb,Tm@CaF_2_:Eu core@shell UCNPs (scale bar = 50 nm); Inset: HAADF-STEM imaging of NaGdF_4_:Yb,Tm@ CaF_2_:Eu core@shell UCNPs (scale bar = 10 nm). c) CaF_2_ shell thickness size distribution. d) NaGdF_4_:Yb,Tm@CaF_2_:Eu core@shell UCNPs EDS mapping of Gd (core) and Eu (shell) (scale bar = 25 nm).


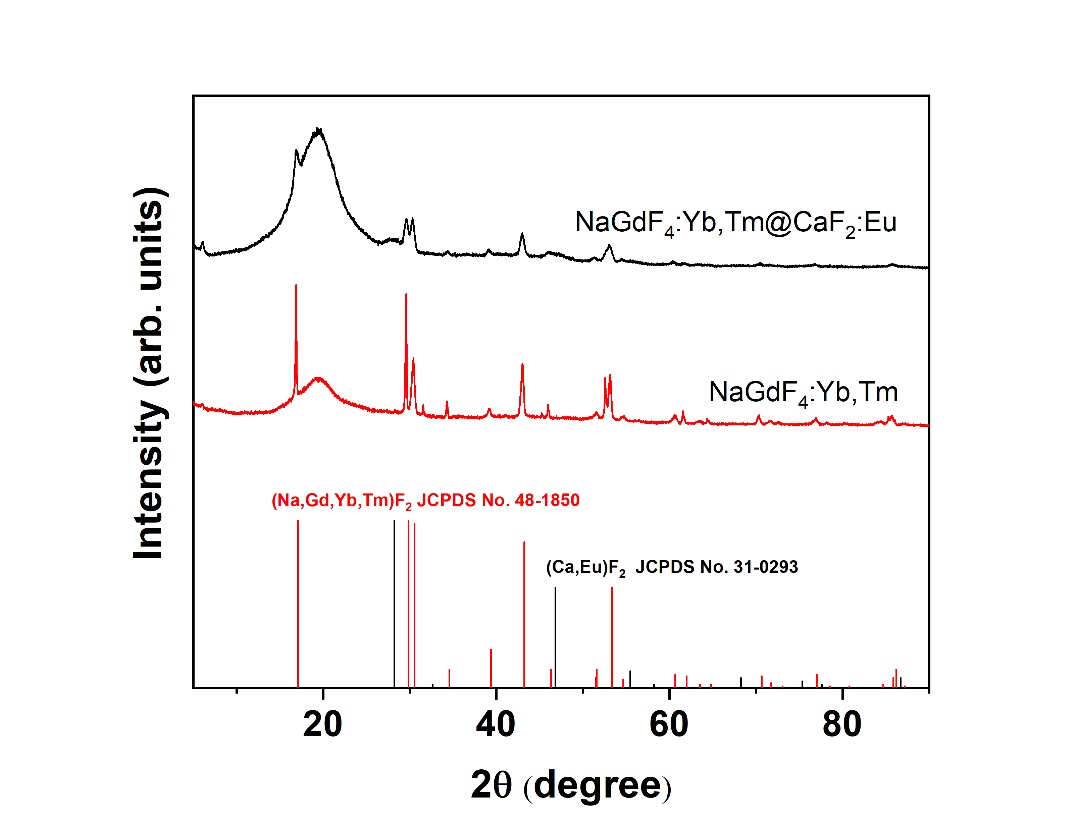


**Supplementary Figure 14:** X-ray diffraction spectrum of NaGdF_4_:Yb,Tm@CaF_2_:Eu UCNPs.


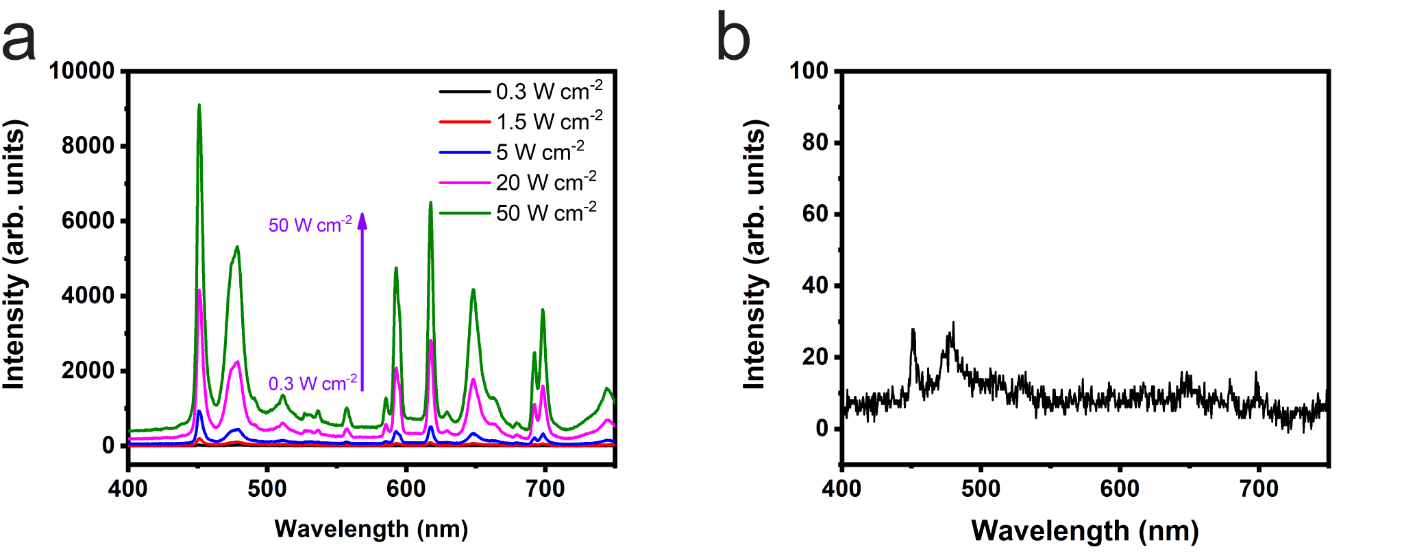


**Supplementary Figure 15:** a) Power-dependent UCL spectra of NaGdF_4_:Yb,Tm@CaF_2_:Eu core@shell UCNP under 980 nm laser irradiation. b) UCL spectra of NaGdF_4_:Yb,Tm@CaF_2_:Eu core@shell UCNP under 980 nm laser irradiation at a power density of 0.3 W cm^-2^.


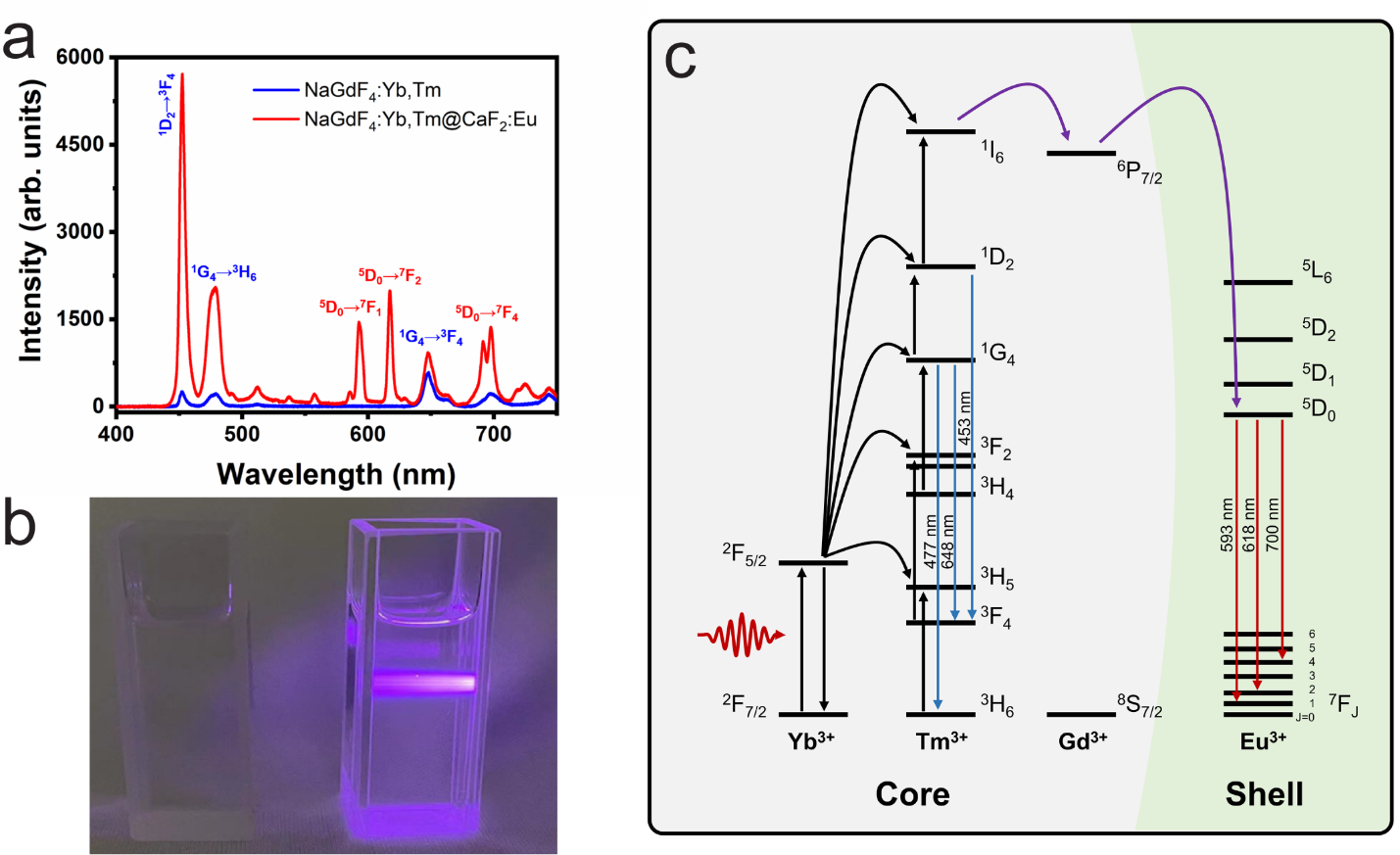


**Supplementary Figure 16:** a) UCL spectra and process of NaGdF_4_:Yb,Tm@CaF_2_:Eu core@shell UCNP and NaGdF_4_:Yb,Tm core UCNP. b) Image of NaGdF_4_:Yb,Tm@CaF_2_:Eu core@shell UCNP (right) and NaGdF_4_:Yb,Tm core UCNP (left) solutions in cyclohexane under 980 nm laser irradiation. c) The energy level configuration of NaGdF_4_:Yb,Tm@CaF_2_:Eu core@shell UCNPs.


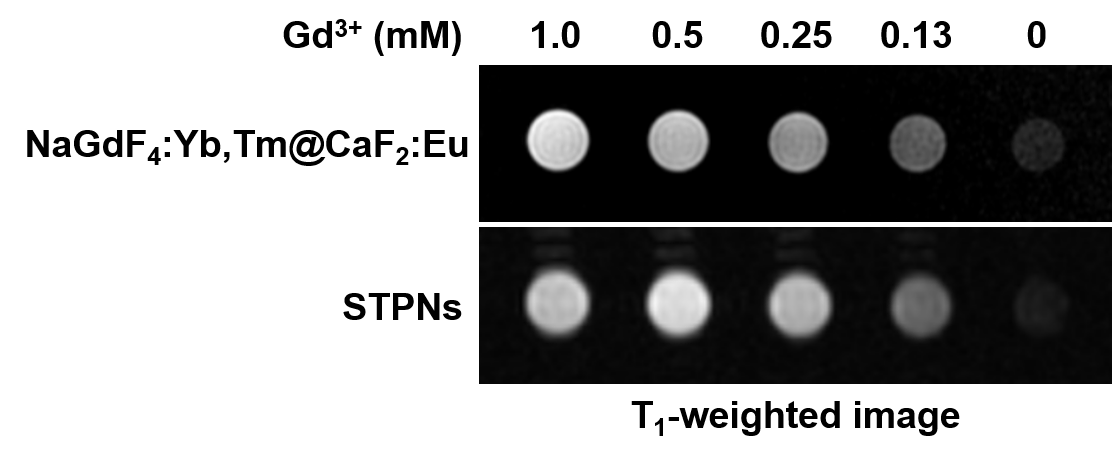


**Supplementary Figure 17:** T_1_-weighted MR images of NaGdF_4_:Yb,Tm@CaF_2_:Eu core@shell UCNP and STPNs.


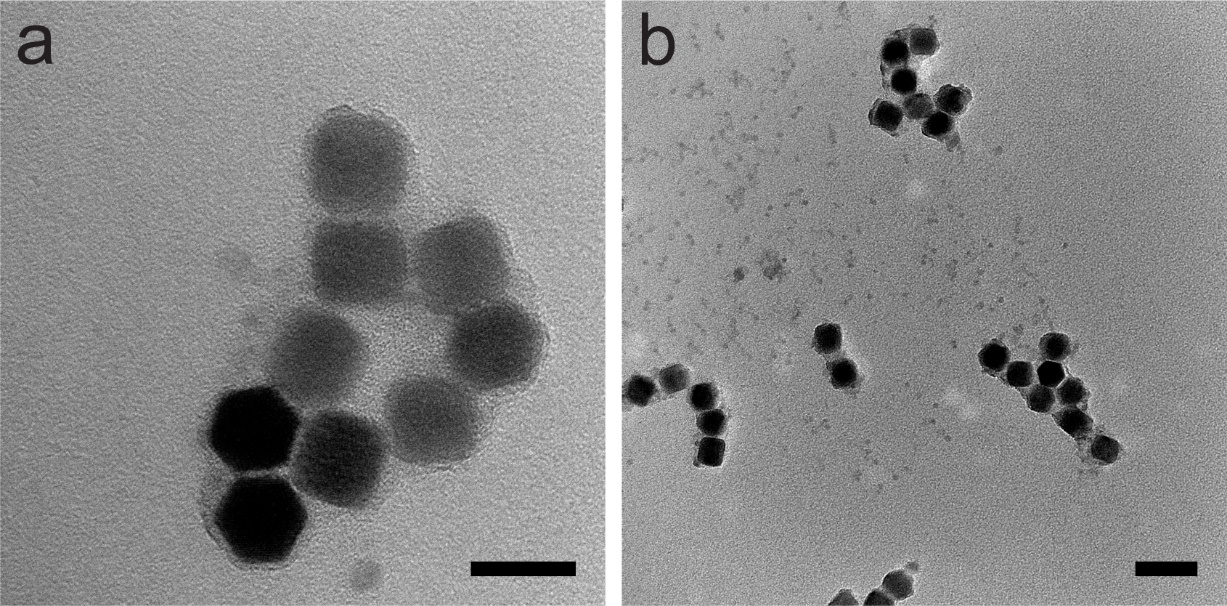


**Supplementary Figure 18:** TEM image of STPNs at a) pH 7.4 (Scale bar = 25 nm) and b) pH 6.5 (Scale bar = 50 nm) (n = 3 independent experiments).





**Supplementary Figure 19:** Changes in absorbance spectra of STPNs at different pH values.


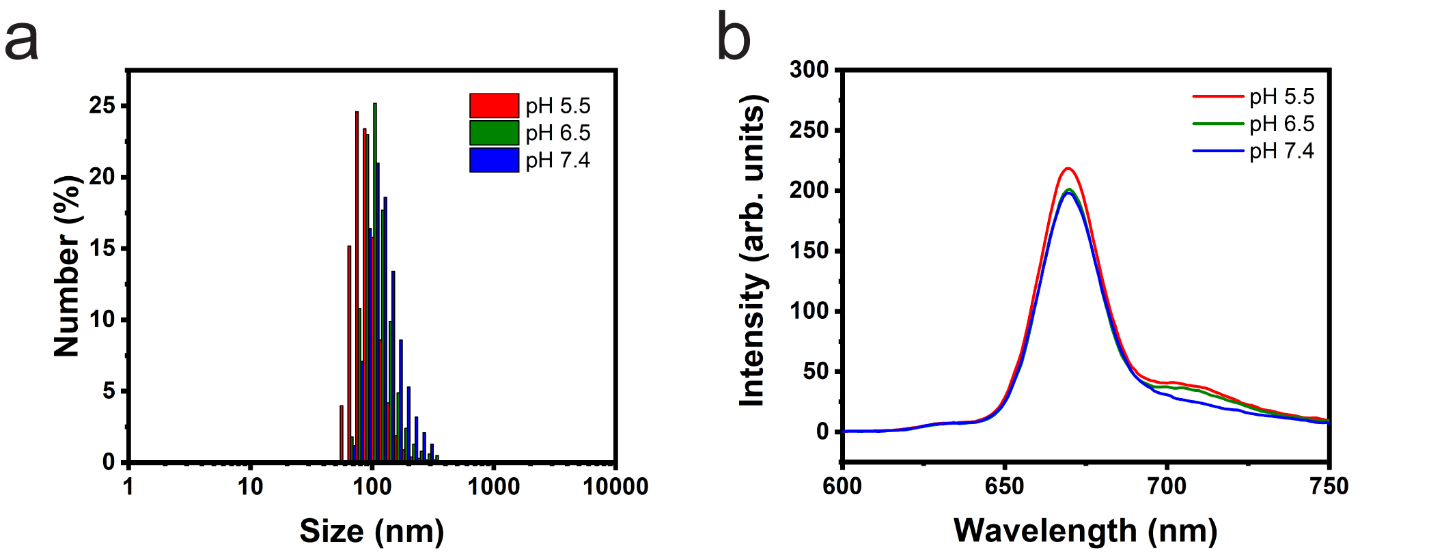


**Supplementary Figure 20:** a) Particle size distribution of SITPNs and b) fluorescence intensity of Pu18 from SITPNs at different pH values.


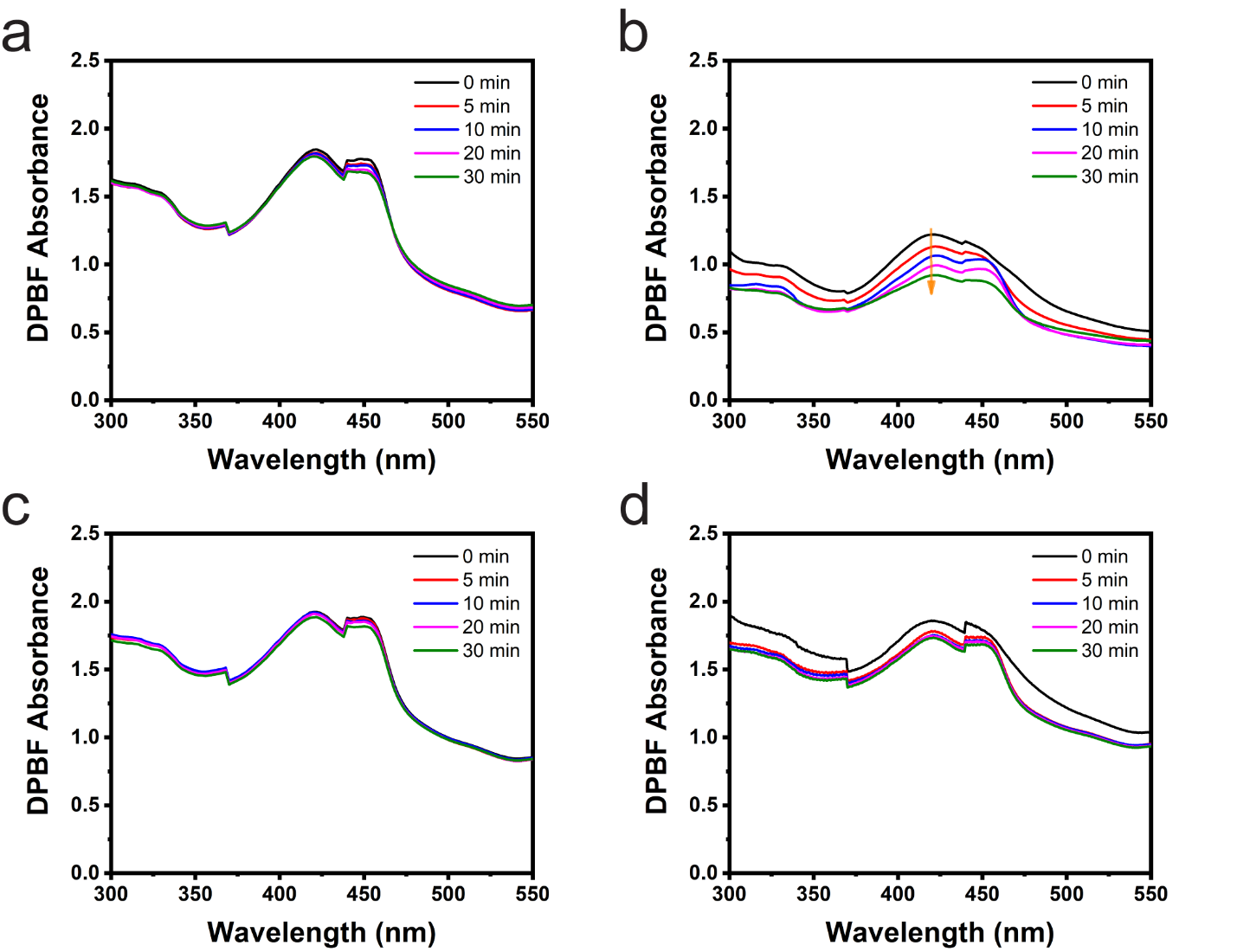


**Supplementary Figure 21:** Changes in DPBF-absorbance spectra of a) STPNs at pH 7.4, b) STPNs at pH 6.5, c) SPLs at pH 7.4, and d) SPLs at pH 6.5 under 980 nm laser irradiation.





**Supplementary Figure 22:** Changes in the DPBF-absorbance spectra of STPNs when pH values fluctuate under 980 nm laser irradiation. It exhibits a negligible decrease at pH 7.4 and a substantial decrease at pH 5.5.


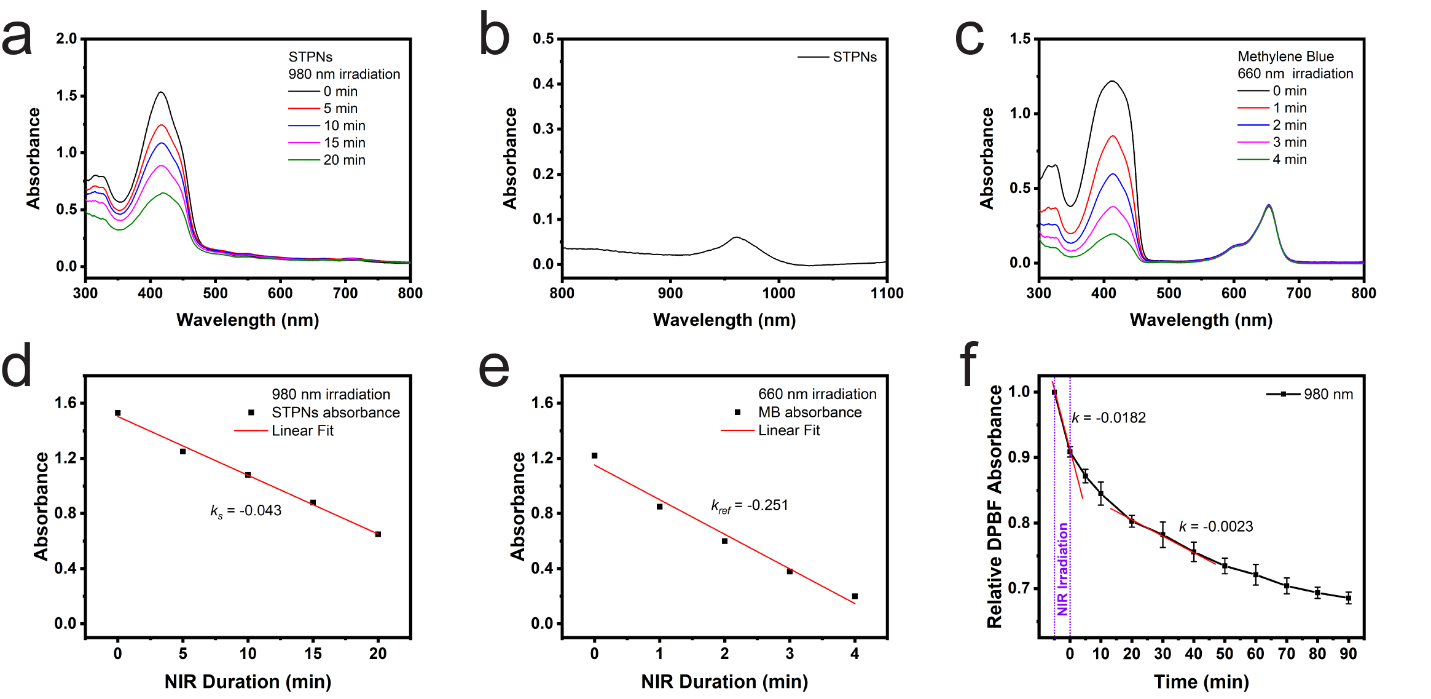


**Supplementary Figure 23:** a) STPNs decrease in DPBF absorbance under continuous 980 nm laser irradiation. b) STPNs absorption spectra at the laser irradiation wavelength region. c) Decrease in absorbance of DPBF in dichloromethane in the presence of reference photosensitizer methylene blue under continuous 660 nm laser irradiation. d) Time-dependent DPBF absorbance decrease at 414 nm in dichloromethane under the presence of STPNs and e) reference photosensitizer methylene blue. f) STPNs decrease in DPBF absorbance after 5 min of 980 nm laser irradiation. The data are represented as mean ± SD (n = 3 independent experiments).


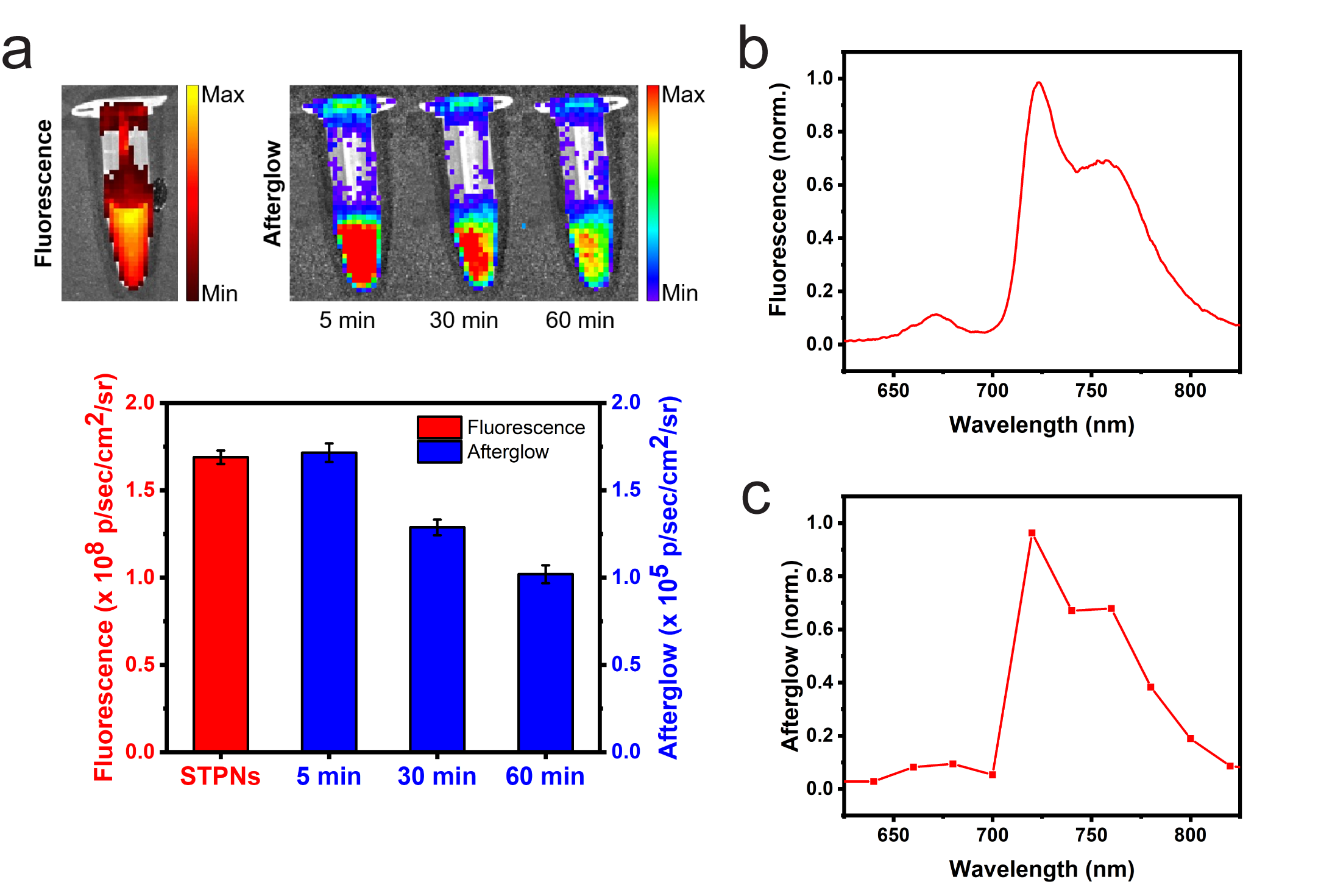


**Supplementary Figure 24:** a) STPNs fluorescence and afterglow images and intensity 5, 30, and 60 min after 980 nm laser irradiation. b) Normalized fluorescence spectra of STPNs under 980 nm laser irradiation. c) Normalized afterglow luminescence spectra of STPNs 30 min after 980 nm laser irradiation. The data are represented as mean ± SD (n = 3 independent experiments).





**Supplementary Figure 25:** ESR spectra of STPNs for ^1^O_2_ generation after 980 nm laser irradiation.


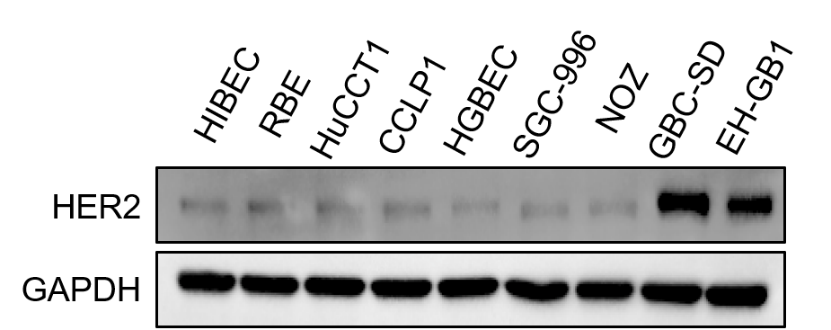


**Supplementary Figure 26:** Western blot for HER2 expression of normal biliary tract tissue, normal gallbladder tissue, and biliary tract tumor cell lines.


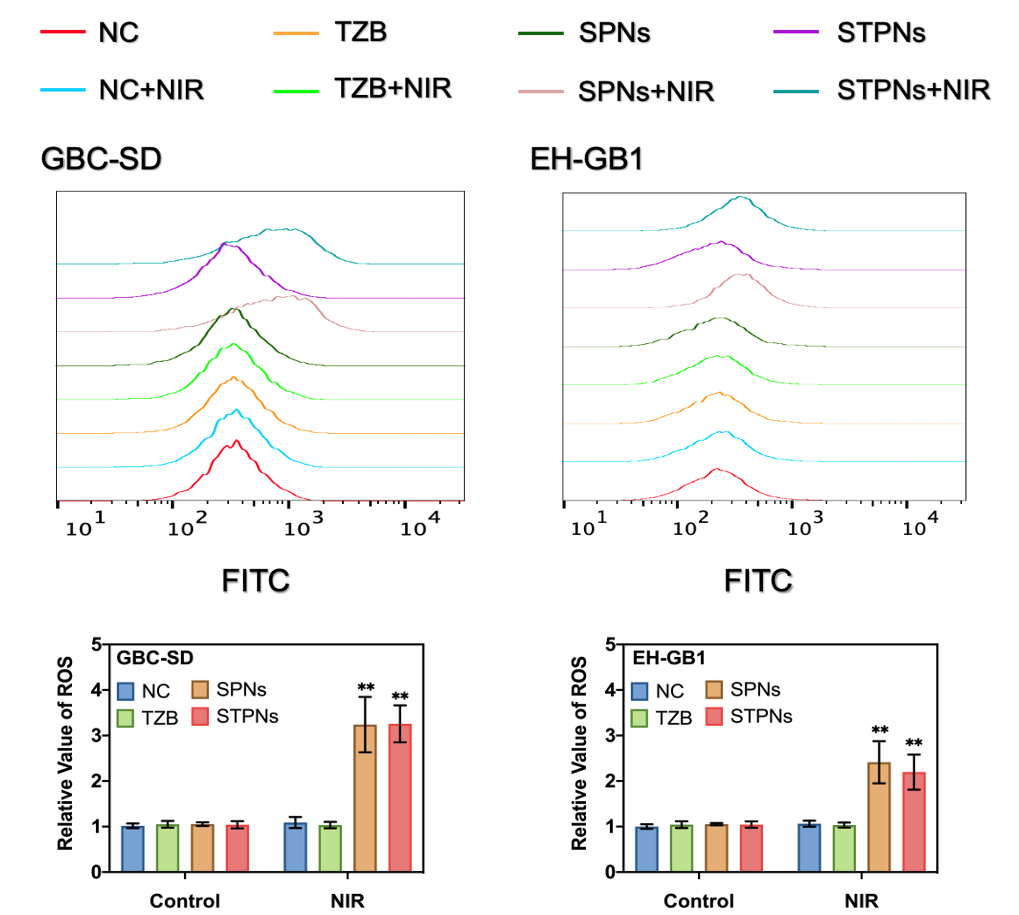


**Supplementary Figure 27:** ROS assays of GBC-SD or EH-GB1 cells exposed to normal media, TZB, SPNs, or STPNS with/without 980 nm laser irradiation (2.0 W cm^-2^, 5 min with every 1 min interval) indicated by flow cytometry. The data are represented as mean ± SD (n = 3 independent experiments). ***P* < 0.01.


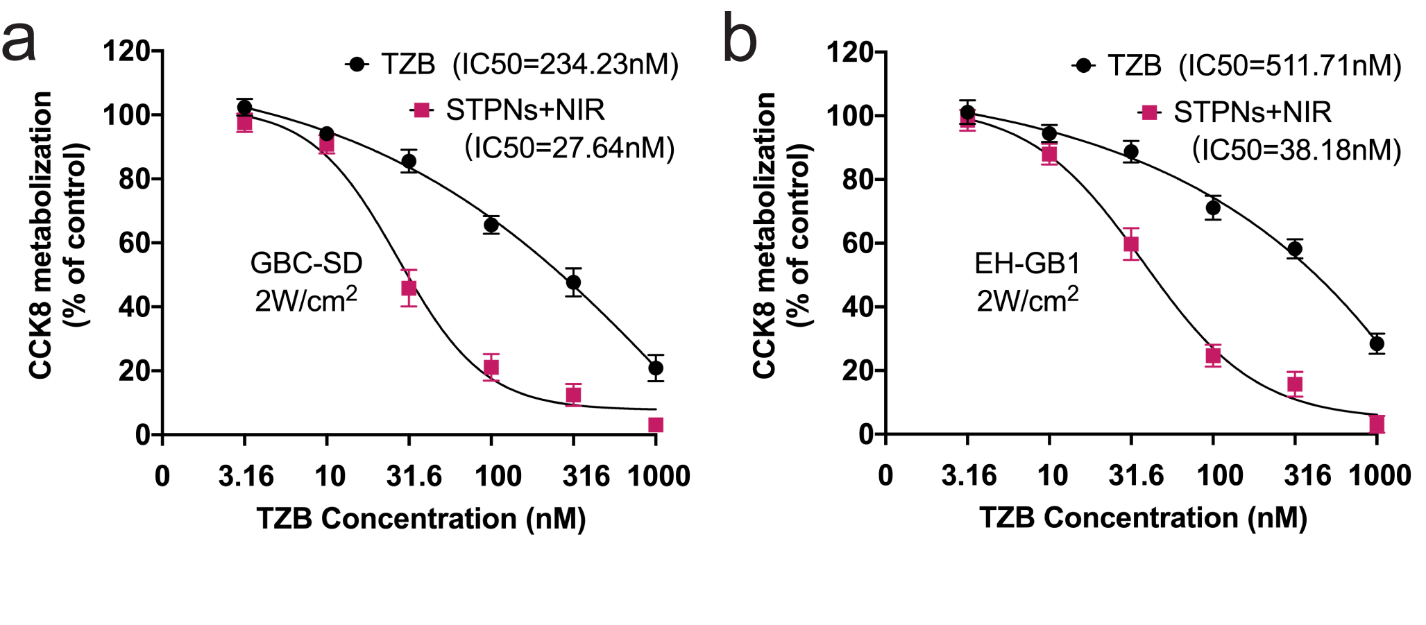


**Supplementary Figure 28:** 48 h half-maximal inhibitory concentration (IC50) curve of a) GBC-SD and b) EH-GB1 cells exposed to TZB or STPNs with 980 nm laser irradiation (2.0 W cm^-2^, 5 min with every 1 min interval). The data are represented as mean ± SD (n = 3 independent experiments).


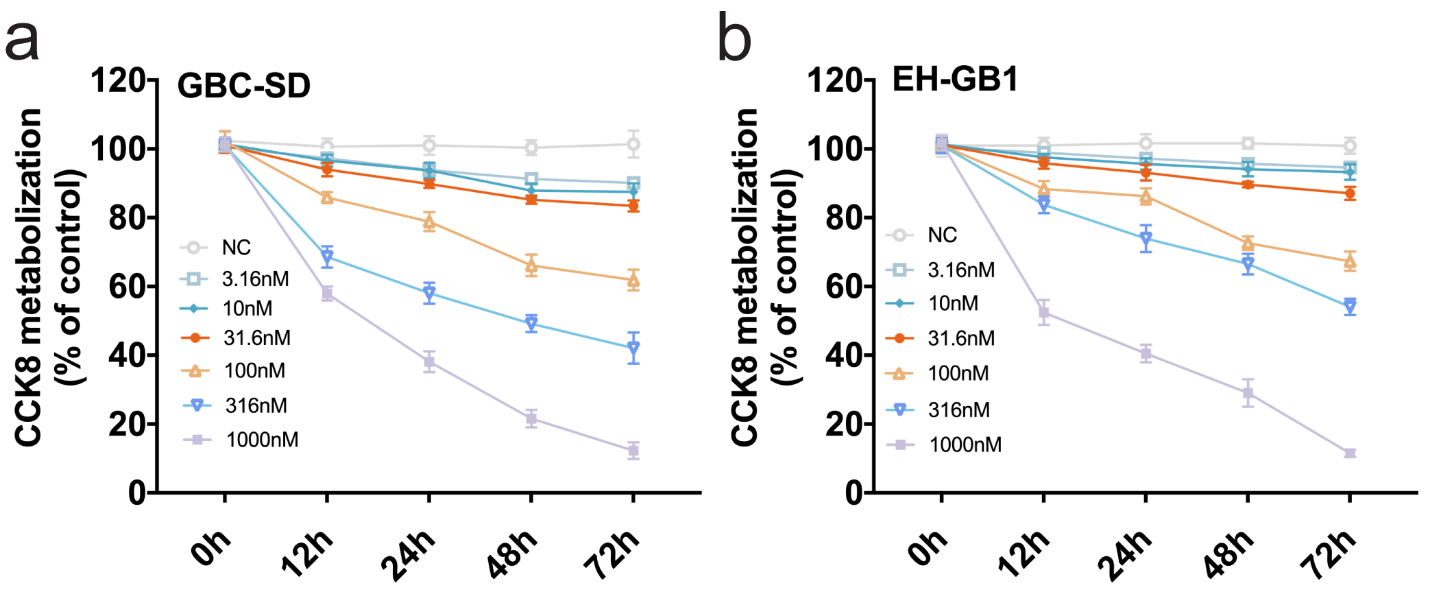


**Supplementary Figure 29:** 0-72 h viability of a) GBC-SD and b) EH-GB1 cells exposed to different concentrations of TZB. The data are represented as mean ± SD (n = 3 independent experiments).


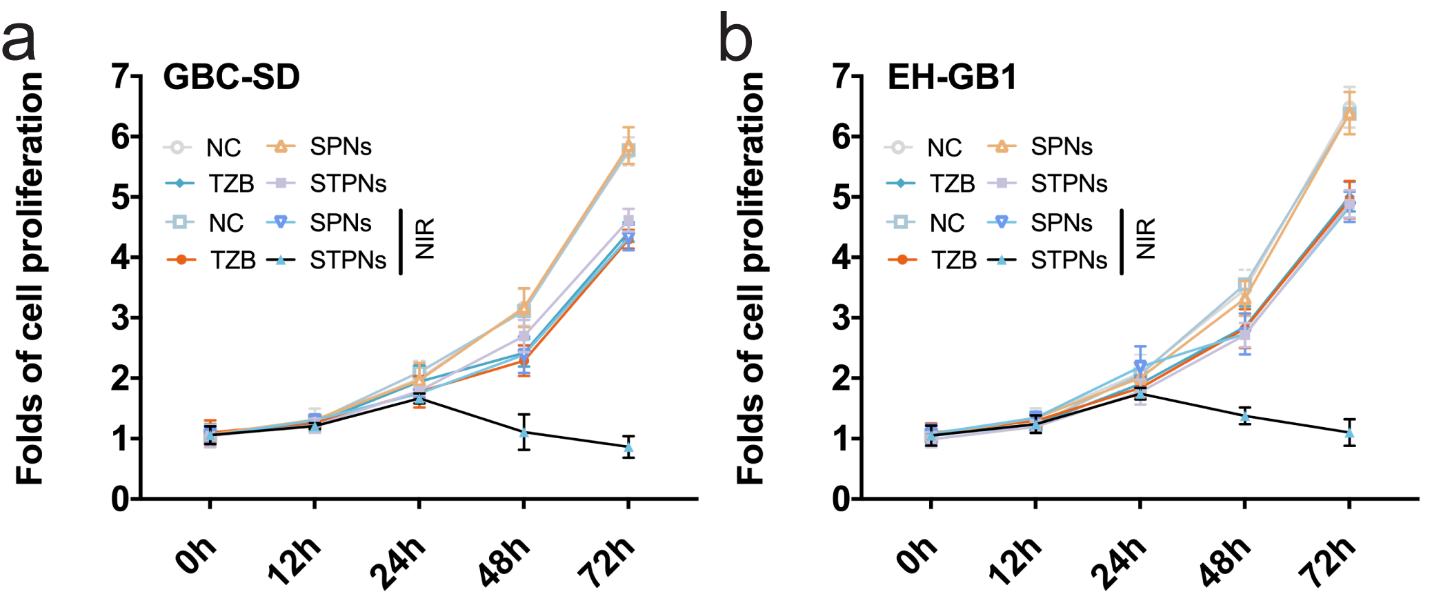


**Supplementary Figure 30:** 0-72 h growth curve of a) GBC-SD and b) EH-GB1 cells exposed to normal media, TZB, SPNs, and STPNs with and without 980 nm laser irradiation (2.0 W cm^-2^, 5 min with every 1 min interval). The data are represented as mean ± SD (n = 3 independent experiments).


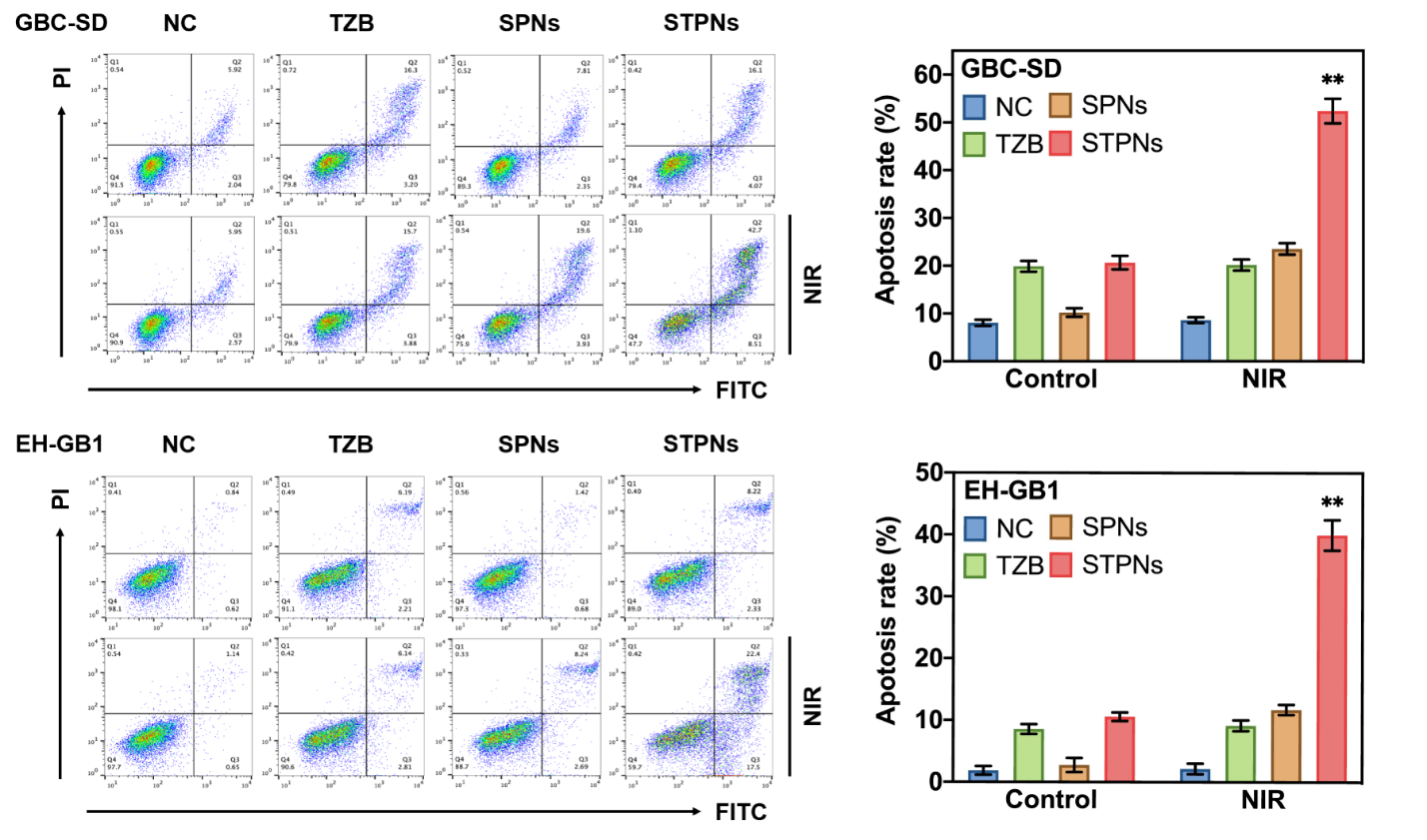


**Supplementary Figure 31:** 48 h apoptosis measurement by flow cytometry of GBC-SD or EH-GB1 cells treated as indicated. The data are represented as mean ± SD (n = 3 independent experiments, one-way ANOVA and Tukey’s multiple comparison test). ***P* < 0.01.


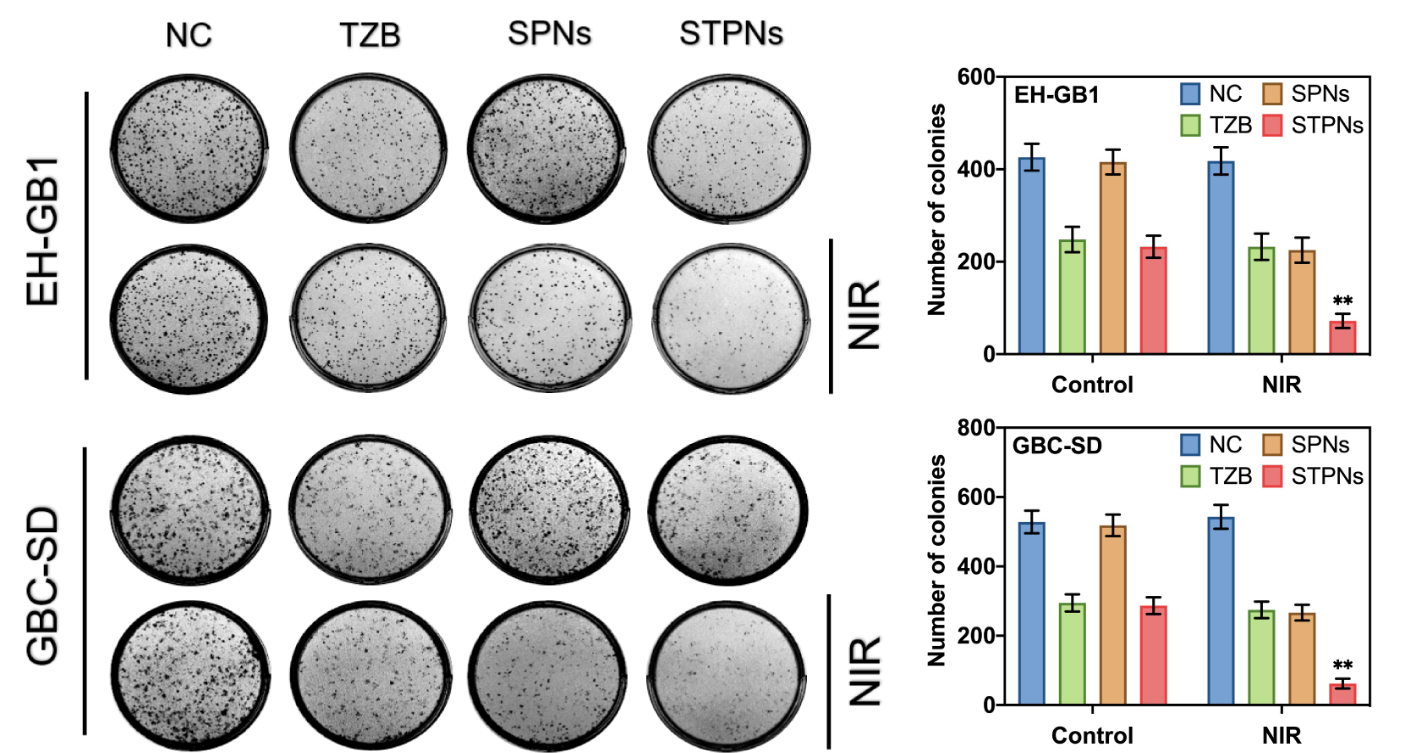


**Supplementary Figure 32:** Representative images of colonies formed by GBC-SD or EH-GB1 cells treated as specified and measurement of colony numbers in treatment groups. The data are represented as mean ± SD (n = 3 independent experiments, one-way ANOVA and Tukey’s multiple comparison test). ***P* < 0.01.


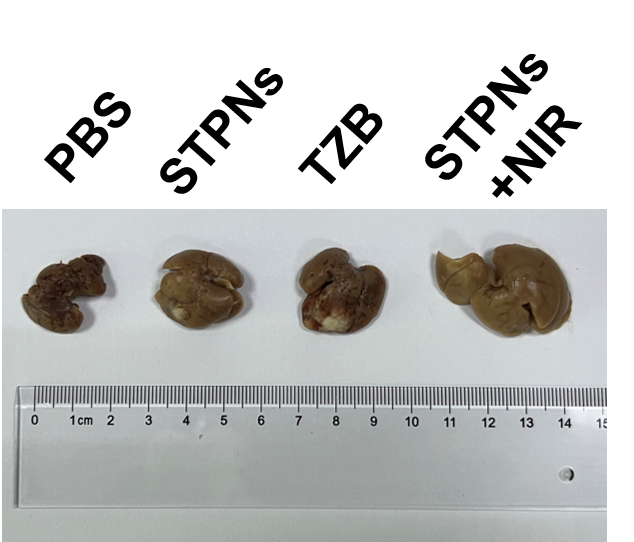


**Supplementary Figure 33:** Representative images of livers and tumors removed from the different groups of mice after treatment.


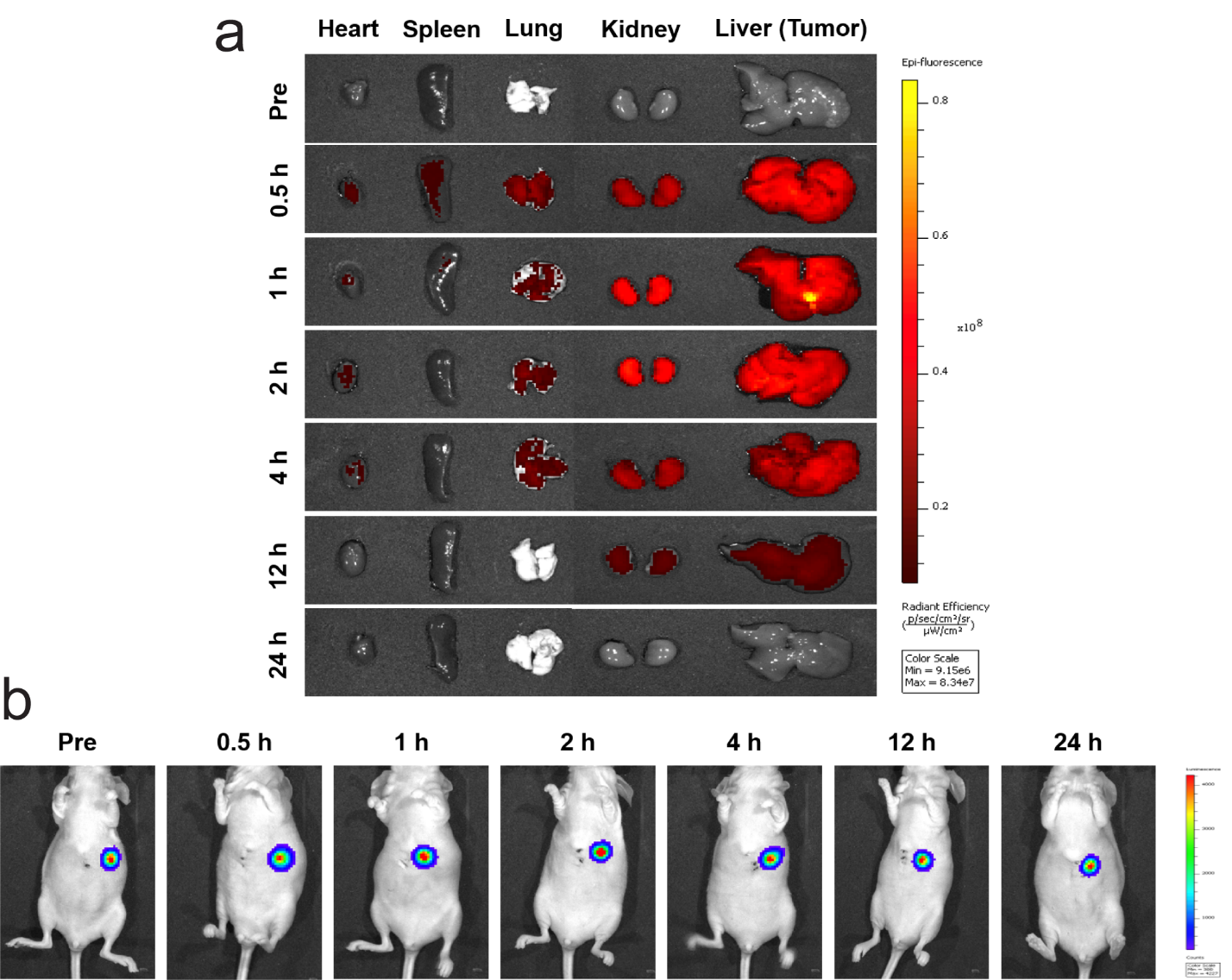


**Supplementary Figure 34:** a) Ex vivo fluorescence (FL) images of livers with their corresponding tumors and other organs collected from mice treated with STPNs after intravenous injection at different time points (0.5, 1, 2, 4, 12, 24 h) and b) in vivo bioluminescence (BL) images of matching orthotopic GBC-bearing mice at different time points.


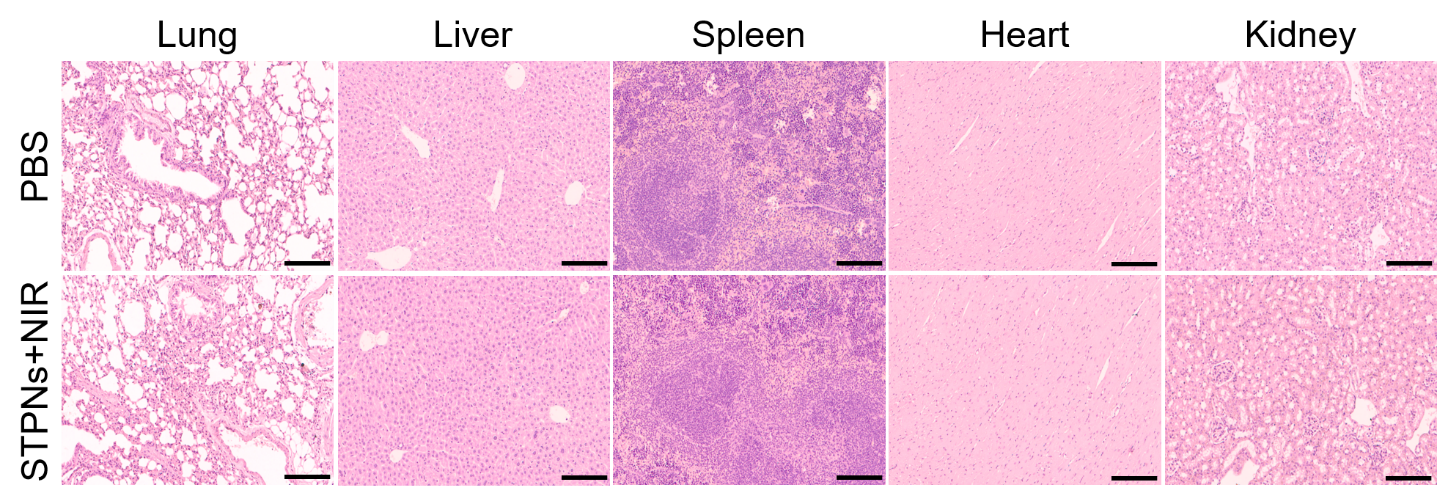


**Supplementary Figure 35:** H&E staining analysis for STPNs in vivo toxicology (scale bar =250 µm).


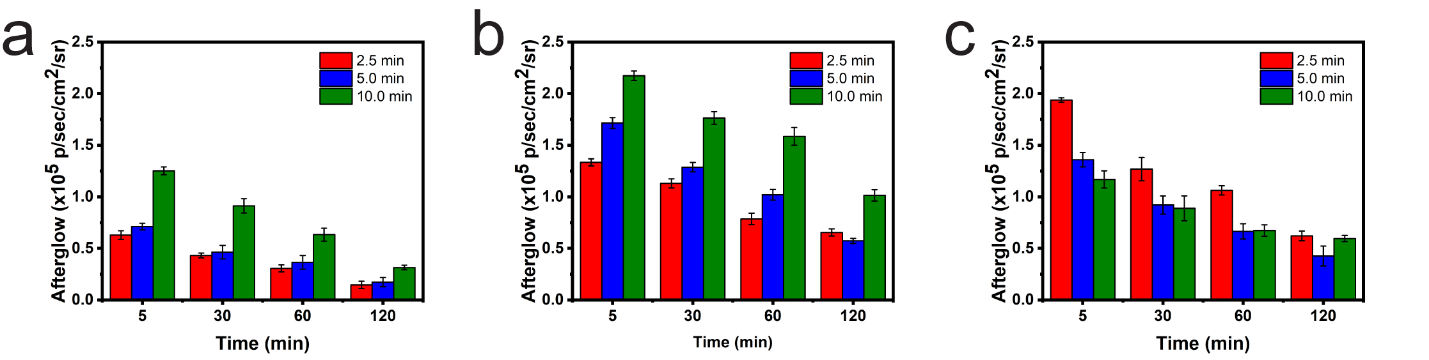


**Supplementary Figure 36:** STPNs afterglow intensity at different irradiation time (2.5, 5, 10 min) under 980 nm laser irradiation at a power of a) 1.0 W cm^-2^, b) 2.0 W cm^-2^, and c) 4.0 W cm^-2^. The data are represented as mean ± SD (n = 3 independent experiments).


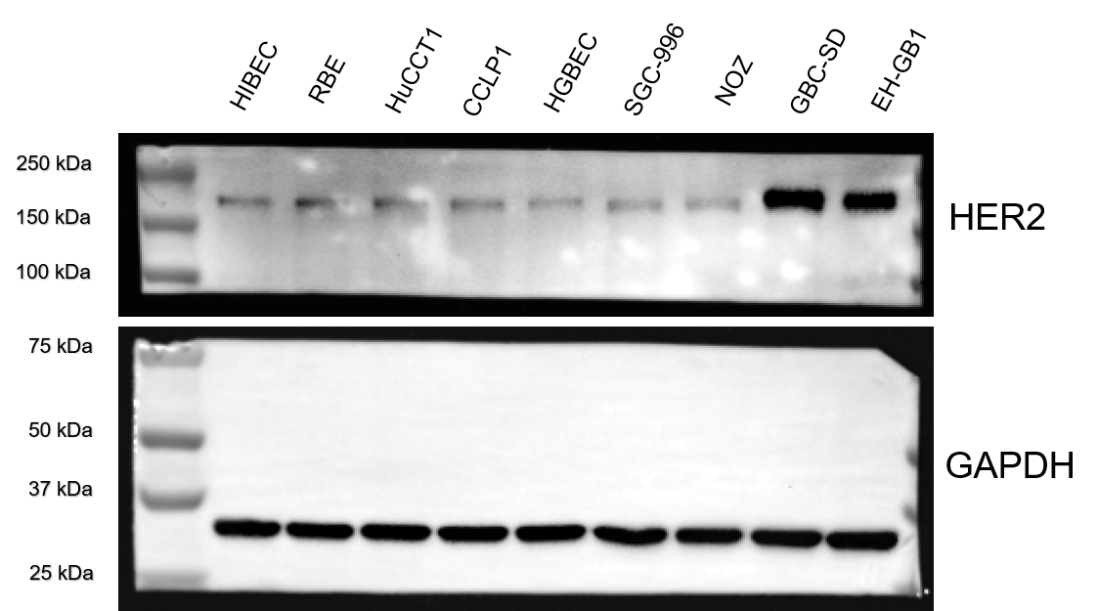


**Supplementary Figure 37:** Uncropped scans of Supplementary Figure 26.


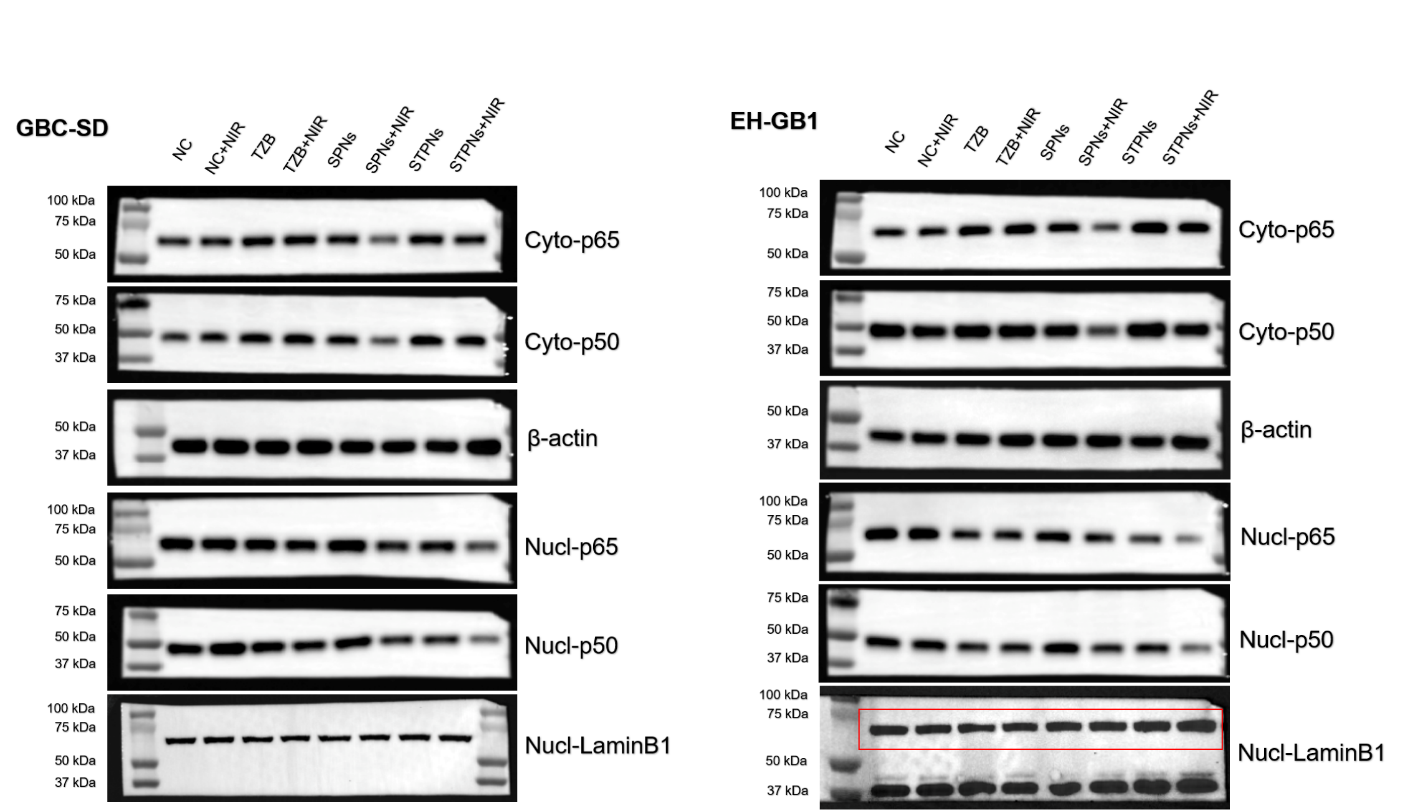


**Supplementary Figure 38:** Uncropped scans of Fig. 4b.


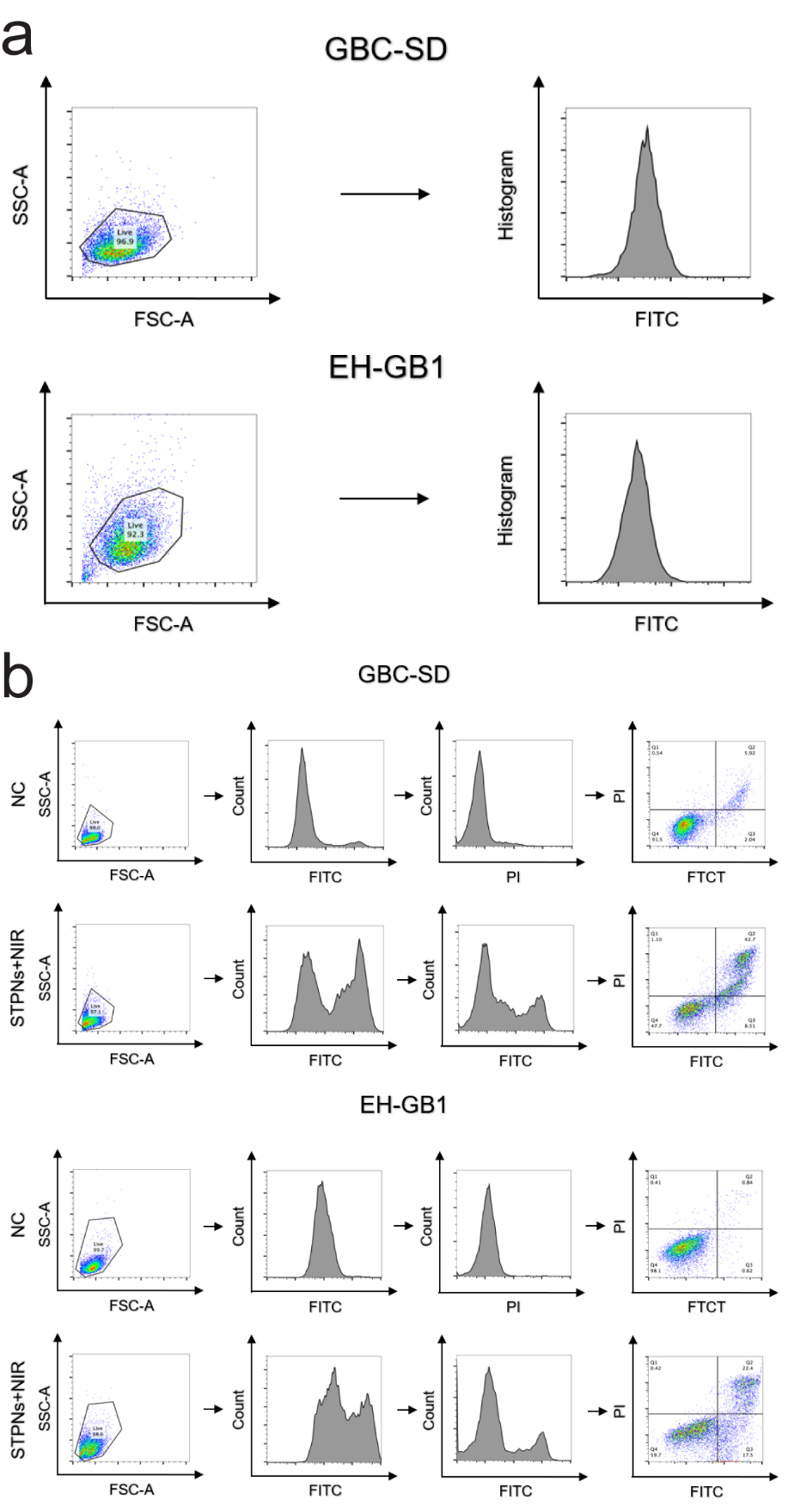


**Supplementary Figure 39:** Gating strategy of the flow cytometry. The gating strategy of flow cytometry analysis for the a) ROS and b) apoptosis in GBC-SD or EH-GB1 cells.
