# Supplementary material for: Pre-activated nanoparticles with persistent luminescence for deep tumor photodynamic therapy in gallbladder cancer
Source: Nat Commun. 2023 Sep 14;14:5699. doi: 10.1038/s41467-023-41389-1 (PMC10502062; doi:10.1038/s41467-023-41389-1)
Supplement: Supplementary file 2 — Reporting Summary [file 41467_2023_41389_MOESM2_ESM.pdf]

nature portfolio

Corresponding author(s): Mingyu Chen, Xiujun Cai, Zhen Gu, Win Topatana

Last updated by author(s): Aug 20, 2023

Reporting Summary

Nature Portfolio wishes to improve the reproducibility of the work that we publish. This form provides structure for consistency and transparency in reporting. For further information on Nature Portfolio policies, see our Editorial Policies and the Editorial Policy Checklist.

Statistics

For all statistical analyses, confirm that the following items are present in the figure legend, table legend, main text, or Methods section.

n/a	Confirmed	
☐	☒	The exact sample size (n) for each experimental group/condition, given as a discrete number and unit of measurement
☐	☒	A statement on whether measurements were taken from distinct samples or whether the same sample was measured repeatedly
☐	☒	The statistical test(s) used AND whether they are one- or two-sided<br>*Only common tests should be described solely by name; describe more complex techniques in the Methods section.*
☒	☐	A description of all covariates tested
☒	☐	A description of any assumptions or corrections, such as tests of normality and adjustment for multiple comparisons
☐	☒	A full description of the statistical parameters including central tendency (e.g. means) or other basic estimates (e.g. regression coefficient) AND variation (e.g. standard deviation) or associated estimates of uncertainty (e.g. confidence intervals)
☐	☒	For null hypothesis testing, the test statistic (e.g. F, t, r) with confidence intervals, effect sizes, degrees of freedom and P value noted<br>*Give P values as exact values whenever suitable.*
☒	☐	For Bayesian analysis, information on the choice of priors and Markov chain Monte Carlo settings
☒	☐	For hierarchical and complex designs, identification of the appropriate level for tests and full reporting of outcomes
☒	☐	Estimates of effect sizes (e.g. Cohen's d, Pearson's r), indicating how they were calculated

Our web collection on statistics for biologists contains articles on many of the points above.

Software and code

Policy information about availability of computer code

Data collection	Zeta potential and DLS data were collected using Zetasizer Software 8.01. FTIR data were collected using Omnic 9.11. Fluorescence data were collected using Andor SOLIS. Absorbance data were collected using Shimadzu LabSolutions. ESR data were collected using WinEPR 4.4. HPLC data were collected using Agilent ChemStation 32.1. Cell fluorescence imaging data were collected using fluorescent microscopy EVOS FL Auto 2. Cryo-TEM cell imaging data were collected using TEM microscopy Talos L120C. Mice bioluminescence and fluorescence imaging data were collected using PerkinElmer IVIS® Lumina LT. Histopathological data were collected using Nanozoomer S60. MR imaging data were collected using 3.0 T MR system. Colony-forming data were collected using BioRadGel DocXR+.
Data analysis	ImageJ was used for image analysis. GraphPad Prism 8.0.1 and Origin 2018 was used to plot the figures and conduct statistical analysis. MestReNova 6.1 was used for H-NMR analysis. MDI Jade 6 was used for XRD analysis. Image Lab 6.0.1 was used for western blot data. FlowJo 10.4 was used to analyze flow cytometry data. Living Image 4.7.3 was used for mice bioluminescence and fluorescence images.

For manuscripts utilizing custom algorithms or software that are central to the research but not yet described in published literature, software must be made available to editors and reviewers. We strongly encourage code deposition in a community repository (e.g. GitHub). See the Nature Portfolio guidelines for submitting code & software for further information.

Data

Policy information about availability of data

All manuscripts must include a data availability statement. This statement should provide the following information, where applicable:

- Accession codes, unique identifiers, or web links for publicly available datasets
- A description of any restrictions on data availability
- For clinical datasets or third party data, please ensure that the statement adheres to our policy

All data generated or analyzed during this study are included in this published article (and its Supplementary Information files). All other data are available from the corresponding authors upon request.

Human research participants

Policy information about studies involving human research participants and Sex and Gender in Research.

Reporting on sex and gender	N/A
Population characteristics	N/A
Recruitment	N/A
Ethics oversight	N/A

Note that full information on the approval of the study protocol must also be provided in the manuscript.

Field-specific reporting

Please select the one below that is the best fit for your research. If you are not sure, read the appropriate sections before making your selection.

[x] Life sciences [ ] Behavioural & social sciences [ ] Ecological, evolutionary & environmental sciences

For a reference copy of the document with all sections, see nature.com/documents/nr-reporting-summary-flat.pdf

Life sciences study design

All studies must disclose on these points even when the disclosure is negative.

Sample size	Details regarding the sample size of all experiments are provided in the figure legends. Sample size was estimated to achieve about 90% power for detection of significant differences in tumor volume between groups based on means and standard deviations in preliminary studies. All available samples passing the quality control were included.
Data exclusions	No data were excluded from analysis.
Replication	Experiments were repeated at least three times with similar results. All attempts at replication were successful.
Randomization	In vitro cell experiments were seeded identically at the onset of the experiments and randomized into the various treatment groups prior to the beginning of treatment protocols.<br>For in vivo studies, once mice were implanted with tumor tissues/cells, they were randomized into experimental groups of 5 mice prior to treatment administration.
Blinding	The investigators and authors have been consistently blinded to the group allocation during data collection and analysis.

Reporting for specific materials, systems and methods

We require information from authors about some types of materials, experimental systems and methods used in many studies. Here, indicate whether each material, system or method listed is relevant to your study. If you are not sure if a list item applies to your research, read the appropriate section before selecting a response.

Materials & experimental systems

n/a	Involved in the study
☐	☒ Antibodies
☐	☒ Eukaryotic cell lines
☒	☐ Palaeontology and archaeology
☐	☒ Animals and other organisms
☒	☐ Clinical data
☒	☐ Dual use research of concern

Methods

n/a	Involved in the study
☒	☐ ChIP-seq
☐	☒ Flow cytometry
☒	☐ MRI-based neuroimaging

Antibodies

Antibodies used	Western blotting: Rabbit monoclonal anti-HER2 (Abcam, EP1045Y, Catalog no.ab134182, 1:1000 dilution), Mouse monoclonal anti-GAPDH (Abcam, 6C5, Catalog no.ab8245, 1:5000 dilution), Rabbit monoclonal anti-NFκB p65 (Abcam, E379, Catalog no.ab32536, 1:10000 dilution), Rabbit monoclonal anti-NFκB p50 (Abcam, E381, Catalog no.ab32360, 1:10000 dilution), Mouse monoclonal anti-beta actin (Abcam, 8226, Catalog no.ab8226, Use a concentration of 1 µg/ml) and Rabbit polyclonal anti-Lamin B1 (Abcam, Nuclear Envelope Marker, Catalog no.ab16048; Use a concentration of 0.1 µg/ml) were obtained from Abcam.<br>Immunohistochemistry staining: Rabbit monoclonal anti-Ki67 (Abcam, EPR3610, Catalog no.ab92742, 1:1000 dilution) was obtained from Abcam.
Validation	Antibodies used were commercially available and all antibodies were validated by manufacturers, with related data shown on the manufacturer website.<br>Anti-HER2, anti-GAPDH, anti-NFκB p65, anti-NFκB p50, anti-beta actin and anti-Lamin B1 were used for relative mechanism validation by western blot experiments on human gallbladder carcinoma cell lines GBC-SD and EH-GB1. Anti-Ki67 were used for validation of nanoparticles tumor-killing effect by immunohistochemistry experiments on orthotopic tumor tissues of mice.

Eukaryotic cell lines

Policy information about cell lines and Sex and Gender in Research

Cell line source(s)	Human gallbladder carcinoma cell line GBC-SD was obtained from the Shanghai Institute for Biological Science, Chinese Academy of Science (Shanghai, China) and human gallbladder carcinoma cell lines EH-GB1 was supplied by Prof. Ying-Bin Liu's lab at Xinhua Hospital Affiliated to Shanghai Jiao Tong University School of Medicine, China. GBC-SD was derived from the gallbladder cancer tissues of a 61-year-old Chinese man. EH-GB1 was derived from the gallbladder cancer tissues of a 53-year-old Chinese woman.
Authentication	Both GBC-SD and EH-GB1 have STR certificates.
Mycoplasma contamination	All cell lines we use are free from mycoplasma contamination.
Commonly misidentified lines (See ICLAC register)	No commonly misidentified cell lines were used in this study.

Animals and other research organisms

Policy information about studies involving animals; ARRIVE guidelines recommended for reporting animal research, and Sex and Gender in Research

Laboratory animals	Female BALB/c nude mice (4 weeks old) were obtained from Shanghai SLAC Laboratory Animal Co., Ltd. All animals were bred in a pathogen-free facility with a 12 h light/dark cycle at 20 ± 3 °C and 40-50% humidity and had ad libitum access to food and water.
Wild animals	This study did not involve wild animals.
Reporting on sex	Theoretically, the findings are applicable to both females and males. Female nude mice were used in this study design because gallbladder cancer is more likely to develop and grow in estrogen-high individuals. Female nude mice are used for better establishment of orthotopic tumor models
Field-collected samples	No field-collected samples were used in this research
Ethics oversight	All animal procedures were performed following the Guidelines for Care and Use of Laboratory Animals of Zhejiang University (Protocol No. 24417).

Note that full information on the approval of the study protocol must also be provided in the manuscript.

Flow Cytometry

Plots

Confirm that:

- [x] The axis labels state the marker and fluorochrome used (e.g. CD4-FITC).
- [x] The axis scales are clearly visible. Include numbers along axes only for bottom left plot of group (a 'group' is an analysis of identical markers).
- [x] All plots are contour plots with outliers or pseudocolor plots.
- [x] A numerical value for number of cells or percentage (with statistics) is provided.

Methodology

Sample preparation	ROS assays: GBC-SD and EH-GB1 cells were seeded in 24-well plates at 100000 per well until adherent and replaced with different media for 12 h incubation. Each cell line was also divided into 8 groups and treated similarly as mentioned previously. 4 hours after irradiation, for flow cytometry analysis, adherent cells of all groups were harvested and washed with PBS 3 times; for observation of the fluorescence cell imager, adherent cells of all groups were washed with PBS 3 times. Next, each group was stained with 500 µL (25 µM) 2,7-dichlorodihydrofluorescein diacetate (Sigma-Aldrich, USA) for 30 min at 37oC in the dark and washed by PBS 3 times. The cells were then immediately analyzed by flow cytometry (BD LSRFortessaTM, USA).<br>PI/Annexin V apoptosis assay: GBC-SD and EH-GB1 cells were seeded in 24-well plates at 100000 per well until adherent and replaced with different media for 12 h incubation. Each cell line was also divided into 8 groups and treated similarly as mentioned previously. After 48 h, all cells in each group, including attached and floating cells, were collected through trypsinization (0.25% Trypsin) without EDTA (Gibco), then washed with PBS. Annexin V-FITC and PI were used to identify apoptotic cells using the Annexin V-FITC Apoptosis Detection Kit I (BD Biosciences) according to the manufacturer's instructions. Viable and dead cells were detected via a flow cytometer (BD LSRFortessaTM, USA).
Instrument	Flow cytometer (BD LSRFortessaTM, USA).
Software	For collection: The software Diva that comes with LSRFortessaDiva ; For analyzation: Flowjo V10.8.1
Cell population abundance	No sorting was performed
Gating strategy	Gating strategy are referred to those described in the BD website. Gating was based on FSC/SCC together with viability dyes and singlet populations. The cell populations within the gate were further analysed based on the expression of markers.

- [x] Tick this box to confirm that a figure exemplifying the gating strategy is provided in the Supplementary Information.
